# Increasing Nutrient Solution pH Alleviated Aluminum-Induced Inhibition of Growth and Impairment of Photosynthetic Electron Transport Chain in* Citrus sinensis* Seedlings

**DOI:** 10.1155/2019/9058715

**Published:** 2019-08-27

**Authors:** Tao-Yu Yang, Li-Ya Cai, Yi-Ping Qi, Lin-Tong Yang, Ning-Wei Lai, Li-Song Chen

**Affiliations:** ^1^Institute of Plant Nutritional Physiology and Molecular Biology, College of Resources and Environment, Fujian Agriculture and Forestry University, Fuzhou 350002, China; ^2^Institute of Materia Medica, Fujian Academy of Medical Sciences, Fuzhou 350002, China; ^3^Fujian Provincial Key Laboratory of Soil Environmental Health and Regulation, College of Resources and Environment, Fujian Agriculture and Forestry University, Fuzhou 350002, China

## Abstract

Although the physiological and molecular responses of* Citrus* to Al-toxicity or low pH have been examined in some details, little information is available on* Citrus* responses to pH and aluminum (Al) interactions.* Citrus sinensis *seedlings were irrigated for 18 weeks with nutrient solution at a concentration of 0 or 1 mM AlCl_3_•6H_2_O and a pH of 2.5, 3.0, 3.5, or 4.0. Thereafter, biomass, root, stem, and leaf concentrations of Al and nutrients, leaf gas exchange, chlorophyll a fluorescence (OJIP) transients, and related parameters were investigated to understand the physiological mechanisms underlying the elevated pH-induced alleviation of* Citrus *toxicity. Increasing the nutrient solution pH from 2.5 to 4.0 alleviated the Al-toxic effects on biomass, photosynthesis, OJIP transients and related parameters, and element concentrations, uptake, and distributions. In addition, low pH effects on the above physiological parameters were intensified by Al-toxicity. Evidently, a synergism existed between low pH and Al-toxicity. Increasing pH decreased Al uptake per root dry weight and its concentration in roots, stems, and leaves and increased nitrogen, phosphorus, calcium, magnesium, sulfur, and boron uptake per plant and their concentrations in roots, stems, and leaves. This might be responsible for the elevated pH-induced alleviation of growth inhibition and the impairment of the whole photosynthetic electron transport chain, thus preventing the decrease of CO_2_ assimilation.

## 1. Introduction

Aluminum (Al) exists mostly as silicate or oxide precipitates that are biologically inactive in neutral or moderately acidic soils. However, Al solubility increases greatly in acidic soils (pH < 5), resulting in the release of phytotoxic Al^3+^ from clay minerals into soil solution [1]. Micromolar concentration of Al^3+^ can cause a rapid inhibition of root elongation and subsequently impair the uptake of water and nutrients, leading to poor growth and yield loss of crops [2]. Therefore, Al-toxicity in acid soils has been regarded as a major factor limiting crop productivity worldwide, since ~ 30% of free ice land is acidic [3]. Furthermore, soil acidity is becoming an increasingly serious problem due to the improper farming practices and environmental deterioration [4]. In recent decades, many researchers have investigated Al-toxic effects on plant growth [5, 6], uptake of nutrients [6, 7], leaf CO_2_ assimilation [8–11], and photosynthetic electron transport [12, 13].

The toxicity of Al to plants depends not only on their Al-tolerance, but also on the soil properties, primarily pH [14]. Hence, it is necessary to investigate the combination effects of Al and pH on plants to better understand the adaption of plants to acidic soils with high active Al. However, such data are very rare, because Al-toxicity and low pH are almost examined separately in different experiments. To our best knowledge, most of studies regarding pH effects on plant Al-toxicity have focused on the pH-induced alterations in toxic Al species and activities in solution, root growth, and root tissue (cell sap) Al concentration, and the results are not consistent [1, 14, 15]. Because Al-toxicity occurs mainly on acidic soils, it is generally believed that the lower the pH in the culture medium, the greater the toxicity of Al to plants. Degenhardt et al. [16] reported that an Al-induced increase in rhizosphere pH was responsible for the Al-resistance in the* Arabidopsis alr*-104 mutant and that increasing the solution pH from 4.4 to 4.5 improved root growth rate of both* alr*-104 mutant and wild type under Al-toxicity. Wang et al. [17] observed that the Al-tolerant wheat cultivar had higher capacity to keep higher rhizosphere pH relative to the Al-sensitive and that increasing the solution pH from 4.5 to 5.0 enhanced the Al-resistance of wheat. Using* Eucalyptus* trees, Yang et al. [18] found that raising the nutrient solution pH from 3.0 to 4.0 increased net photosynthesis and transpiration under Al-toxicity. However, Kinraide [19] found that the activities of Al^3+^ in soil solutions peaked at ~ pH 4.1 in the range of pH 3.5 to 5.5, implying that Al-toxicity in soils with pH 4.1 might be more severe than that in more acidic or alkaline soils. Al level in rice root cell sap increased as solution pH increased from 4.0 to 6.0 [20].

*Citrus* often display poor growth and a shortened lifetime in low pH soils with high active Al [21]. In China,* Citrus* are cultivated mainly on acidic soils. Moreover, soil acidity is becoming an increasing urgent problem in some* Citrus* orchards [22]. Although the physiological and molecular responses of* Citrus* to Al-toxicity or low pH have been investigated in some details [9, 23–25], very scarcity is known on* Citrus* responses to pH and Al interactions. Preliminary study showed that increasing nutrient solution pH prevented Al-toxic effects on* Citrus* growth and photosynthesis. Here, we used* C. sinensis* seedlings as materials and investigated the effects of pH and Al interactions on biomass; Al and nutrient elements in roots, stems, and leaves; gas exchange, chlorophyll (Chl) a fluorescence (OJIP) transients and related parameters in leaves. Our objective was to determine the physiological mechanisms underlying the increased pH-induced alleviation of* Citrus* Al-toxicity.

## 2. Materials and Methods

### 2.1. Culture and Treatments of Seedlings

Seedling culture and treatments were conducted according to Guo et al. [26] with some modifications. Five-week-old “Xuegan” (*C. sinensis*) seedlings were transplanted to 6L pots (two per pot) containing sand thoroughly washed with tap water, and then planted in a greenhouse under a natural photoperiod at Fujian Agriculture and Forestry University, Fuzhou with an annual average relative humidity, temperature, and sunlight of ~ 76%, 20°C and 1600 h, respectively. Six weeks after transplanting, each pot was replenished every day with freshly prepared nutrient solution [i.e., macronutrients (in mM): Ca(NO_3_)_2_,1; KNO_3_, 1; MgSO_4_, 0.5; KH_2_PO_4_, 0.1; and micronutrients (in *μ*M): Fe-EDTA, 20; ZnSO_4_, 2; MnCl_2_, 2; CuSO_4_, 0.5; and (NH_4_)_6_Mo_7_O_24_, 0.065] at a concentration of 0 or 1 mM AlCl_3_•6H_2_O and a pH of 2.5, 3.0, 3.5 or 4.0 for 18 weeks until a portion of nutrient solution began to leak out from a hole at the bottom of the pot (~ 500 mL). The pH of the solution was adjusted by NaOH or HCl before irrigation. At the end of the experiment, fully expanded (~ 7-week-old) leaves were used for the assays of all physiological parameters.

### 2.2. Measurements of Root, Stem, and Leaf Dry Weights

At the end of the experiment, ten seedlings per treatment from different pots were harvested and then divided into roots, stems, and leaves. Their dry weight (DW) was weighted after being dried to a constant weight at 70°C.

### 2.3. Measurements of Leaf OJIP Transients and Calculation of Related Parameters

Leaf OJIP transients were measured in 3 h dark-adapted seedlings at room temperature using a Handy Plant Efficiency Analyzer (Handy PEA, Hansatech Instruments Limited, Norfolk, UK). All fluorescence parameters were calculated according to Jiang et al. [27] and Banks [28].

### 2.4. Leaf Gas Exchange

Leaf gas exchange was measured with a CIRAS-2 portable photosynthesis system (PP systems, Herts, UK) at a leaf temperature of ~ 26°C, a controlled light intensity of ~ 1000 *μ*mol m^−2^ s^−1^ and a controlled CO_2_ concentration of ~ 380 *μ*mol mol^−1^ between 9 and 12 a.m. on a sunny day. The flow rate through the 2.5 cm^2^ leaf chamber was kept at 200 *μ*mol s^−1^. Water use efficiency (WUE) was calculated as CO_2_ assimilation/transpiration rate.

### 2.5. Assays of Elements, Calculation of Element Uptake, and Distributions in Roots, Stems, and Leaves

The small first- and second-order fibrous roots (< 2.0 mm diameter), the middle sections of stems, and the recent fully expanded mature (~ 7-week-old) leaves were used for the measurements of elements [26]. Al was assayed with a NexION 300X Inductively Coupled Plasma Mass Spectrometer (ICP-MS, PerkinElmer, CT, USA). Phosphorus (P) was measured colorimetrically as blue molybdate-phosphate complexes. Potassium (K) was assayed with a FP640 Flame Photometry (Shanghai Precision Scientific Instrument Co., Ltd., Shanghai, China). Calcium (Ca), magnesium (Mg), iron (Fe), manganese (Mn), copper (Cu), and zinc (Zn) were determined with a PinAAcle 900F Atomic Absorption Spectrometer (Perkinelmer Singapore Pte Ltd., Singapore). Sulfur (S) was determined by the simple turbidimetric method. Nitrogen (N) was assayed by the Kjeldahl method with a Kjeltec 8200 Auto Distillation (FOSS Analytical AB, Höganäs, Sweden). Boron (B) was determined by the curcumin method [22, 24].

Element uptake and distributions were calculated as described previously [26].

### 2.6. Data Analysis

There were 12 pots (24 seedlings) per treatment in a completely randomized design. Results represented the mean ± SE of 4-12 replicates (one plant from different pot per treatment) except for the mean OJIP transients and the different expressions derived from the mean transients. Significant differences among the eight treatments were analysed by four (pH values) × two (Al levels) ANOVA and followed by the least significant difference (LSD) at* P*< 0.05 level.

Principal component analysis (PCA) was performed using a SPSS® statistical software (version 17.0, IBM, NY, USA) [26].

## 3. Results

### 3.1. Seedling Growth

**Figure 1:**
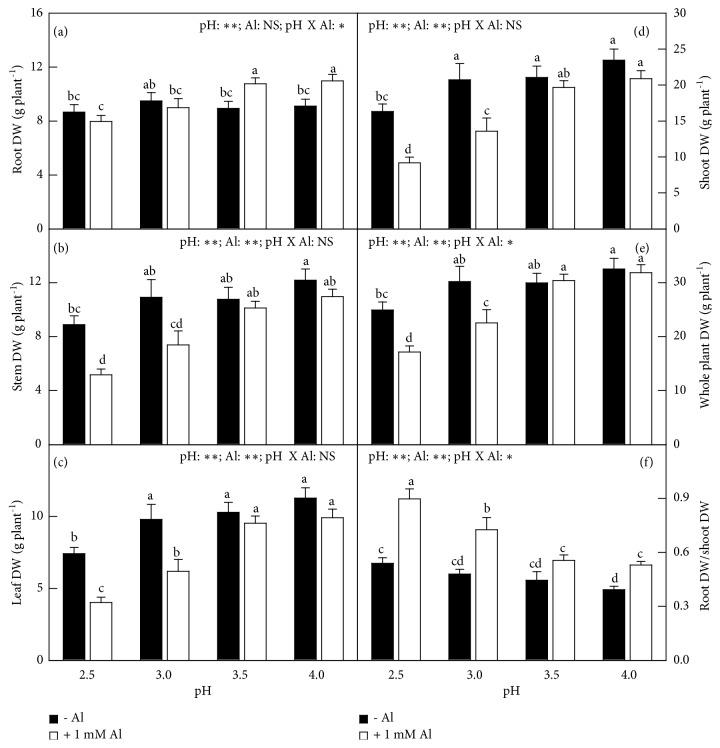
Effects of pH and Al interactions on (a) root, (b) stem, (c) leaf, (d) shoot, (e) whole plant DW, and (f) root DW/shoot DW ratio in* C. sinensis* seedlings. Bars represent means ± SE (*n* = 10). Different letters above the bars indicate a significant difference at* P *< 0.05. NS, *∗* and *∗∗* indicate nonsignificant and significant at 5% and 1% level, respectively.

Without Al-toxicity, only pH 2.5 decreased stem, leaf, shoot, and whole plant DW and increased root DW/shoot DW ratio relative to control (pH 4.0); with Al-toxicity, all the six parameters kept stable when pH decreased from 4.0 to 3.5, then root, stem, leaf, shoot, and whole plant DW decreased and root DW/shoot DW ratio increased with further decreasing pH. Al decreased or did not alter stem, leaf, shoot, and whole plant DW, but it increased or did not affect root DW and root DW/shoot DW ratio. The interactive effects of pH and Al were significant for root DW, whole plant DW, and root DW/shoot DW ratio. Many rotten and died fibrous roots were observed in the pH 2.5 + 1 mM Al-treated seedlings (Figure 1 and [Supplementary-material supplementary-material-1]).

### 3.2. Leaf Gas Exchange

**Figure 2:**
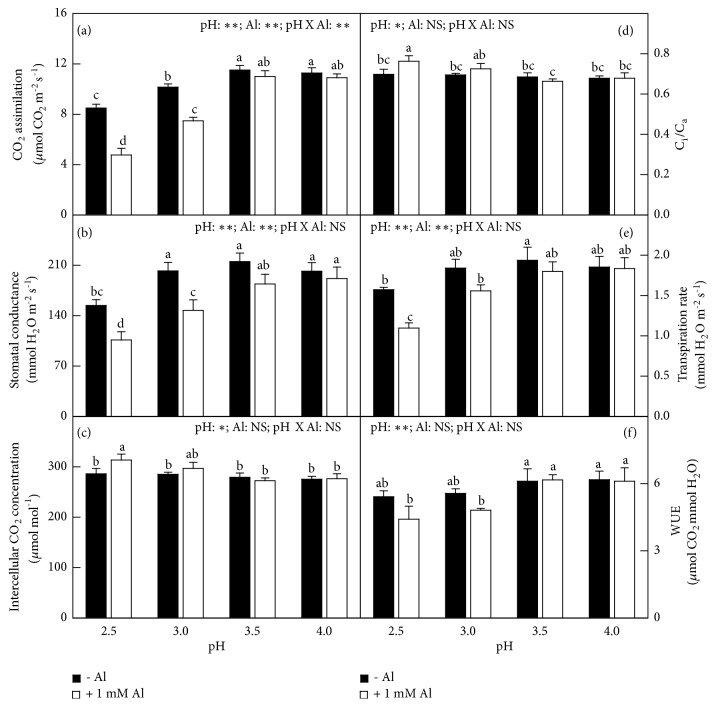
Effects of pH and Al interactions on (a) CO_2_ assimilation, (b) stomatal conductance (g_s_), (c) intercellular CO_2_ concentration (C_i_), (d) ratio of intercellular to ambient CO_2_ concentration (C_i_/C_a_), (e) transpiration rate, and (f) WUE in* C. sinensis* leaves. Bars represent means ± SE (*n* = 5). Different letters above the bars indicate a significant difference at* P *< 0.05. NS, *∗* and *∗∗* indicate nonsignificant and significant at 5% and 1% level, respectively.

Without Al-toxicity, leaf stomatal conductance (g_s_) and transpiration rate (CO_2_ assimilation) did not change as pH decreased from 4.0 to 3.0 (3.5) and then decreased with further decreasing pH, but intercellular CO_2_ concentration (C_i_), the ratio of intercellular to ambient CO_2_ concentration (C_i_/C_a_), and WUE did not alter in response to pH. With Al-toxicity, leaf CO_2_ assimilation, g_s_, transpiration rate, and WUE kept unchanged as pH decreased from 4.0 to 3.5 and then decreased with further decreasing pH, but both C_i_ and C_i_/C_a_ ratio were the highest at pH 2.5. Interactions between pH and Al on leaf gas exchange were significant only for CO_2_ assimilation (Figure 2).

Regression analysis showed that CO_2_ assimilation increased with increasing g_s_, but it increased with decreasing C_i_ and C_i_/C_a_ ratio in leaves (Figure S2).

### 3.3. Leaf OJIP Transients and Related Parameters

**Figure 3:**
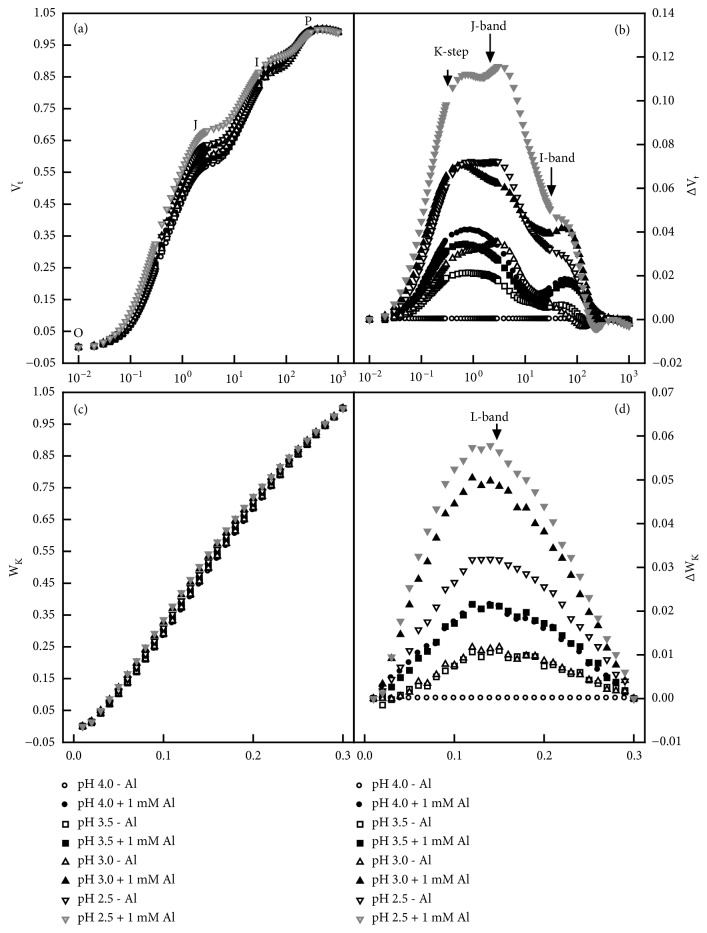
Effects of pH and Al interactions on the mean OJIP transients expressed as the kinetics of relative variable fluorescence: (a) between F_o_ and F_m_: V_t_ = (F_t_ – F_o_)/(F_m_ – F_o_) and (b) the differences of the eight samples to the reference sample submitted to pH 4.0 + 0 mM Al (ΔV_t_); (c) between F_o_ and F_300*μ*s_: W_K_ = (F_t_ – F_o_)/(F_300*μ*s_ – F_o_) and (d) the differences of the six samples to the reference sample submitted to pH 4.0 + 0 mM Al (ΔW_K_).

OJIP transients from the 0 mM Al-treated leaves displayed little alterations in response to pH except for a slight increase in O-step at pH 2.5 compared with OJIP transients from the pH 4.0 + 0 mM Al-treated leaves. Al increased the heterogeneity of samples, especially at pH 2.5. OJIP transients from the 1 mM Al-treated leaves displayed an increased O-step at pH 2.5-3.0 and a suppressed P-step at pH 2.5 compared with OJIP transients from the pH 4.0 + 1 mM Al-treated leaves ([Supplementary-material supplementary-material-1]). OJIP transients from the low pH and/or Al-treated leaves had positive ΔL-, ΔK-, ΔJ-, and ΔI-bands around 150 *μ*s, 300 *μ*s, 2 ms, and 30 ms compared with OJIP transients from the pH 4.0 + 0 mM Al-treated leaves, respectively. The positive ΔL-, ΔK-, ΔJ-, and ΔI-bands were the most pronounced from the leaves treated by pH 2.5 + 1 mM Al (Figure 3).

**Figure 4:**
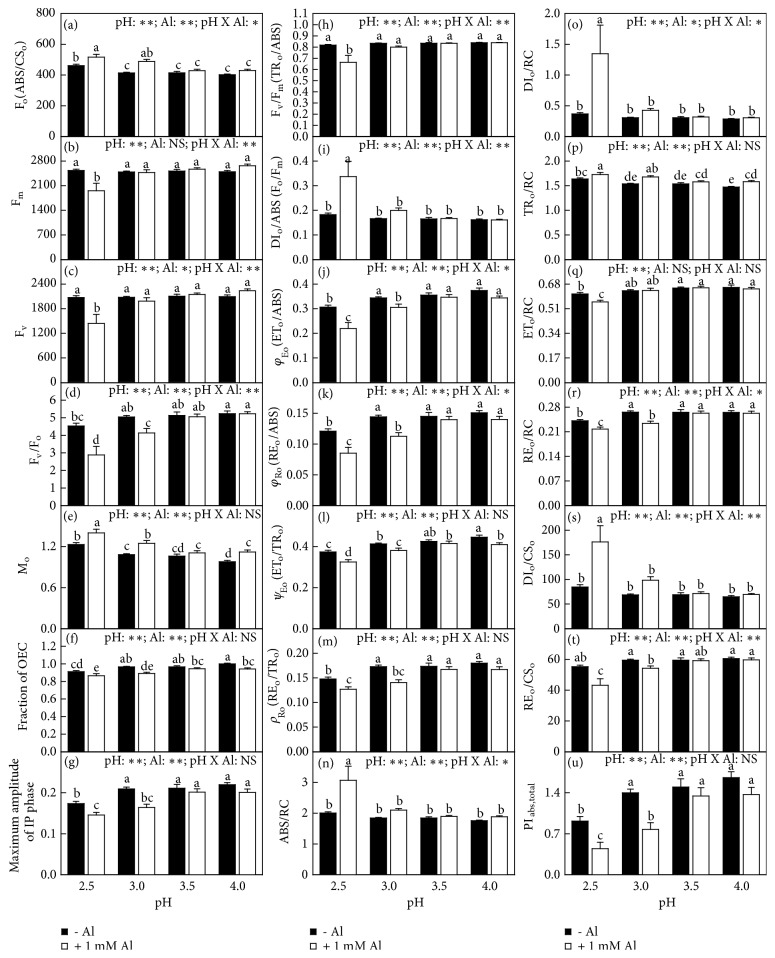
Effects of pH and Al interactions on 21 Chl a fluorescence parameters in dark-adapted* C. sinensis *leaves. Bars represent means ± SE (*n* = 12). Different letters above the bars indicate a significant difference at* P *< 0.05. NS, *∗* and *∗∗* indicate nonsignificant and significant at 5% and 1% level, respectively.

Without Al-toxicity, minimum fluorescence (F_o_), maximum fluorescence (F_m_), maximum variable fluorescence (F_v_), maximum primary yield of photochemistry of photosystem II (PSII; F_v_/F_o_), fraction of oxygen evolving complex (OEC) relative to control (F_OEC_), maximum amplitude of IP phase, maximum PSII efficiency of dark-adapted leaves (F_v_/F_m_), quantum yield for energy dissipation (DI_o_/ABS), quantum yield for electron transport (ET_o_/ABS), quantum yield for the reduction of end acceptors of PSI per photon absorbed (RE_o_/ABS), efficiency with which a trapped exciton can move an electron into the electron transport chain from Q_A_^−^ to the PSI end electron acceptors (RE_o_/TR_o_), absorption flux per reaction centre (RC, ABS/RC), dissipated energy flux per RC (DI_o_/RC), electron transport flux per RC (ET_o_/RC), reduction of end acceptors at PSI electron acceptor side (RE_o_/RC), dissipated energy flux per cross section (CS, DI_o_/CS_o_), reduction of end acceptors at PSI electron acceptor side (RE_o_/CS_o_), and total performance index (PI_abs,total_) displayed little change in response to pH except for increased F_o_ and TR_o_/RC and decreased F_v_/F_o_, F_OEC_, maximum amplitude of IP phase, ET_o_/ABS, RE_o_/ABS, RE_o_/TR_o_, ET_o_/RC, RE_o_/RC, and PI_abs,total_ at pH 2.5. However, approximated initial slope (in ms^−1^) of the fluorescence transient V = f(t) (M_o_) and trapped energy flux per RC (TR_o_/RC) increased, and probability that a trapped exciton moves an electron into the electron transport chain beyond Q_A_^−^ (*ψ*_Eo_ or ET_o_/TR_o_) decreased with decreasing pH (Figure 4).

With Al-toxicity, DI_o_/ABS, ABS/RC, DI_o_/RC, and DI_o_/CS_o_ (F_o_, M_o_, and TR_o_/RC) did not change as pH decreased from 4.0 to 3.0 (3.5) and then increased with further decreasing pH. By contrast, F_v_/F_o_, F_OEC_, maximum amplitude of IP phase, ET_o_/ABS, RE_o_/ABS, ET_o_/TR_o_, RE_o_/TR_o_, RE_o_/RC, RE_o_/CS_o_, and PI_abs,total_ (F_m_, F_v_, F_v_/F_m_ and ET_o_/RC) did not change as pH decreased from 4.0 to 3.5 (3.0) and then decreased with further decreasing pH (Figure 4).

Al-toxicity increased or did not affect F_o_, M_o_, DI_o_/ABS, ABS/RC, DI_o_/RC, TR_o_/RC, and DI_o_/CS_o_ but decreased or did not alter the other fluorescence parameters. Interactions between pH and Al on Chl a fluorescence parameters were significant only for F_o_, F_m_, F_v_, F_v_/F_o_, F_v_/F_m_, DI_o_/ABS, ET_o_/ABS, RE_o_/ABS, ABS/RC, DI_o_/RC, RE_o_/RC, and DI_o_/CS_o_ (Figure 4).

Leaf CO_2_ assimilation increased with increasing F_m_, F_v_, F_v_/F_o_, F_OEC_, maximum amplitude of IP phase, F_v_/F_m_, ET_o_/ABS, RE_o_/ABS, ET_o_/TR_o_, RE_o_/TR_o_, ET_o_/RC, RE_o_/RC, RE_o_/CS_o_, or PI_abs,total_, respectively, but it decreased with increasing F_o_, M_o_, DI_o_/ABS, ABS/RC, DI_o_/RC, TR_o_/RC, or DI_o_/CS_o_, respectively ([Supplementary-material supplementary-material-1]).

### 3.4. Concentrations, Uptake, and Distributions of Elements

**Figure 5:**
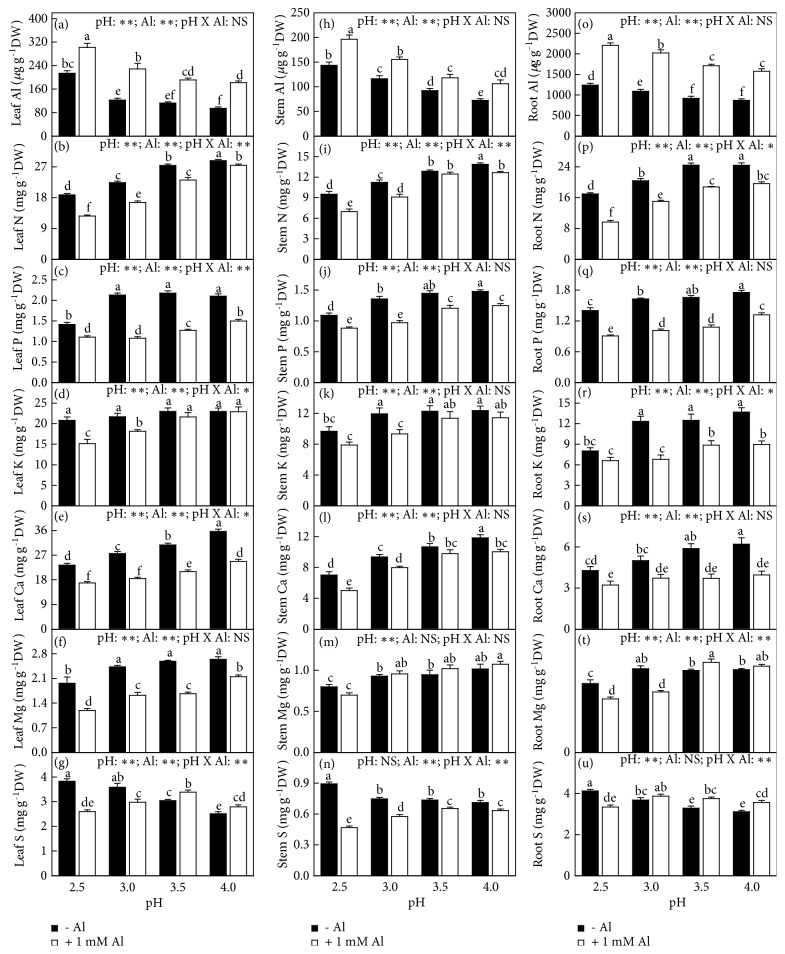
Effects of pH and Al interactions on concentrations of (a, h, o) Al, (b, i, p) N, (c, j, q) P, (d, k, r) K, (e, l, s) Ca, (f, m, t) Mg, and (g, n, u) S in* C. sinensis *(a-g) leaves, (h-n) stems, and (o-u) roots. Bars represent means ± SE (*n* = 8 except for 4 for K and Mg). Different letters above the bars indicate a significant difference at* P *< 0.05. NS, *∗* and *∗∗* indicate nonsignificant and significant at 5% and 1% level, respectively.

**Figure 6:**
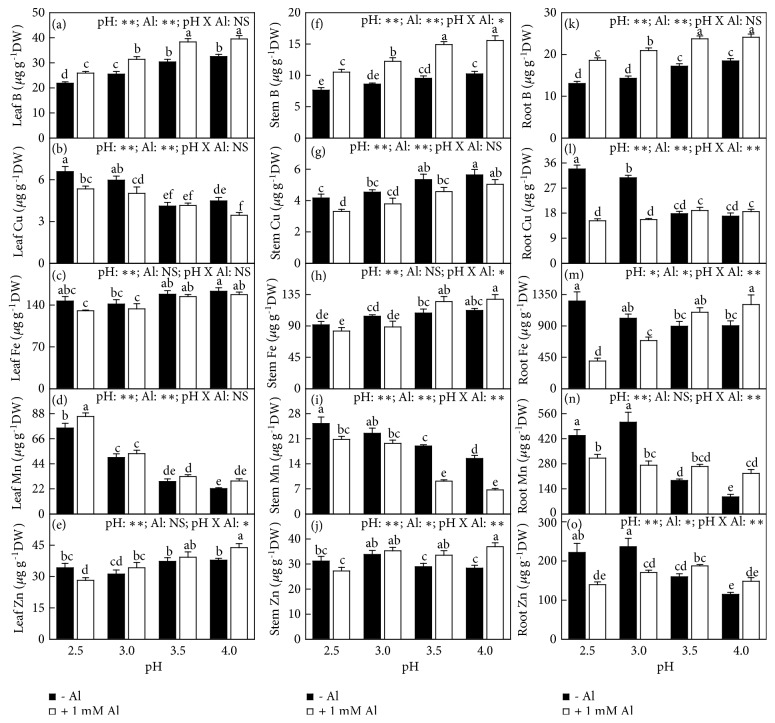
Effects of pH and Al interactions on concentrations of (a, f, k) B, (b, g, l) Cu, (c, h, m) Fe, (d, i, n) Mn, and (e, j, o) Zn in* C. sinensis *(a-e) leaves, (f-j) stems, and (k-o) roots. Bars represent means ± SE (*n* = 8 except for 4 for Fe and Mn). Different letters above the bars indicate a significant difference at* P *< 0.05. NS, *∗* and *∗∗* indicate nonsignificant and significant at 5% and 1% level, respectively.

Without Al-toxicity, the levels of Al, S, and Mn in leaves, stems, and roots, Cu in leaves and roots, Zn in stems and leaves, and Fe in roots increased or did not change with decreasing pH, but the levels of P, N, Ca, K, Mg, and B in leaves, stems, and roots, Fe in leaves and stems, Cu in stems, and Zn in leaves decreased or did not alter with decreasing pH (Figures 5-6).

With Al-toxicity, the levels of Al and Mn in leaves, stems, and roots, and Cu in leaves increased or did not change with decreasing pH. The levels of N, P, K, Ca, Mg, B, and Fe in leaves, stems, and roots, Cu in stems and roots, Zn in leaves and stems, and S in stems decreased or did not change with decreasing pH. The levels of S in leaves and Zn in roots (S in roots) increased as pH decreased from 4.0 to 3.5 (3.0) and then decreased with further decreasing pH (Figures 5-6).

Al-toxicity increased or did not alter the levels of Al and B in leaves, stems, and roots, Zn in leaves and stems, Mn in leaves, and S in roots at each given pH with the exceptions that the levels of Zn in leaves and S in roots were decreased by Al at pH 2.5, but decreased or did not alter the levels of N, P, K, Ca, Mg, Cu, and Fe in leaves, stems, and roots, S and Mn in stems, and Zn in roots with the exception that root Fe level was increased by Al at pH 4.0. The levels of S in leaves and Mn in roots were decreased by Al at pH 2.5-3.0, but increased or unaffected by Al at pH 3.5-4.0. Interactions between pH and Al on element levels were significant only for N, S, Mn, and Zn levels in leaves, stems, and roots, K level in leaves and roots, Fe level in stems and roots, P and Ca levels in leaves, B level in stems, and Mg, and Cu levels in roots (Figures 5-6).

**Figure 7:**
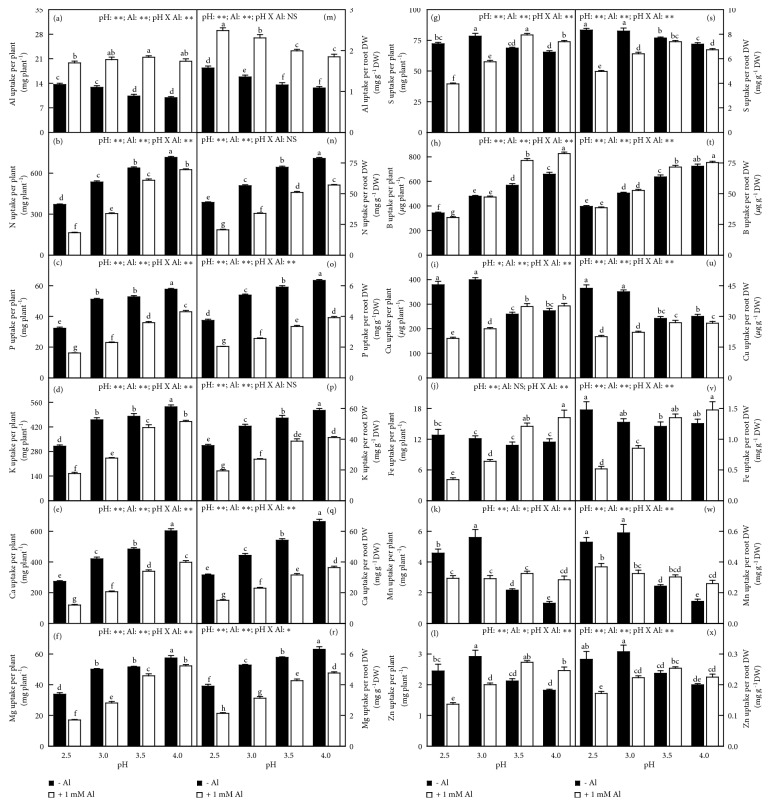
Effects of pH and Al interactions on element uptake (a-l) per plant and (m-x) per root DW in* C. sinensis *seedlings. Bars represent means ± SE (*n* = 8 except for 4 for K, Mg, Fe, and Mn). Different letters above the bars indicate a significant difference at* P *< 0.05. NS, *∗* and *∗∗* indicate nonsignificant and significant at 5% and 1% level, respectively.

Without Al-toxicity, the uptake of Al, S, Cu, Fe, Mn, and Zn per plant (root DW) increased or did not alter with decreasing pH with the exceptions that the uptake of Mn and Zn per plant was higher at pH 3.0 than that at pH 2.5, but the uptake of the other elements per plant (root DW) decreased or kept unchanged with decreasing pH. With Al-toxicity, the uptake of Al and Mn per plant (root DW) increased or did not alter with decreasing pH with the exception that the uptake of Mn per plant was slightly higher at pH 3.5 than that at pH 2.5, but the uptake of the other elements per plant (root DW) decreased or kept unchanged with decreasing pH with the exception that the uptake of S per plant (root DW) was higher at pH 3.5 than that at pH 4.0. Al-toxicity increased the uptake of Al per plant (root DW) and increased or did not alter the uptake of B per plant (root DW) with the exception that the uptake of B per plant was decreased by Al at pH 2.5, but it decreased the uptake of N, P, K, Ca, and Mg per plant (root DW) and decreased or did not change the uptake of S, Cu, Fe, and Zn per root DW. The uptake of S, Cu, Fe, Mn, and Zn per plant and of Mn per root DW was decreased by Al at pH 2.5-3.0, but increased or unaltered by Al at pH 3.5-4.0. Interactions between pH and Al on the uptake of elements were significant for the uptake of all elements except for the uptake of Al, N, and K per root DW (Figure 7).

Whole plant (root) DW increased with increasing uptake of N, P, K, Ca, Mg, S, B, or Fe (S, B, or Fe) per plant ([Supplementary-material supplementary-material-1]).

Leaf CO_2_ assimilation decreased with increasing leaf Al or Mn concentration, but it increased with increasing leaf N, K, Ca, Mg, Fe, or Zn concentrations. Leaf CO_2_ assimilation increased with increasing uptake of N, P, K, Ca, Mg, S, B, or Fe per plant ([Supplementary-material supplementary-material-1]).

Leaf Al increased with increasing F_o_, M_o_, DI_o_/ABS, ABS/RC, DI_o_/RC, TR_o_/RC, or DI_o_/CS_o_, respectively, but it decreased with increasing F_m_, F_v_, F_v_/F_o_, F_OEC_, maximum amplitude of IP phase, F_v_/F_m_, ET_o_/ABS, RE_o_/ABS, ET_o_/TR_o_, RE_o_/TR_o_, ET_o_/RC, RE_o_/RC, RE_o_/CS_o_, or PI_abs,total_, respectively ([Supplementary-material supplementary-material-1]).

**Figure 8:**
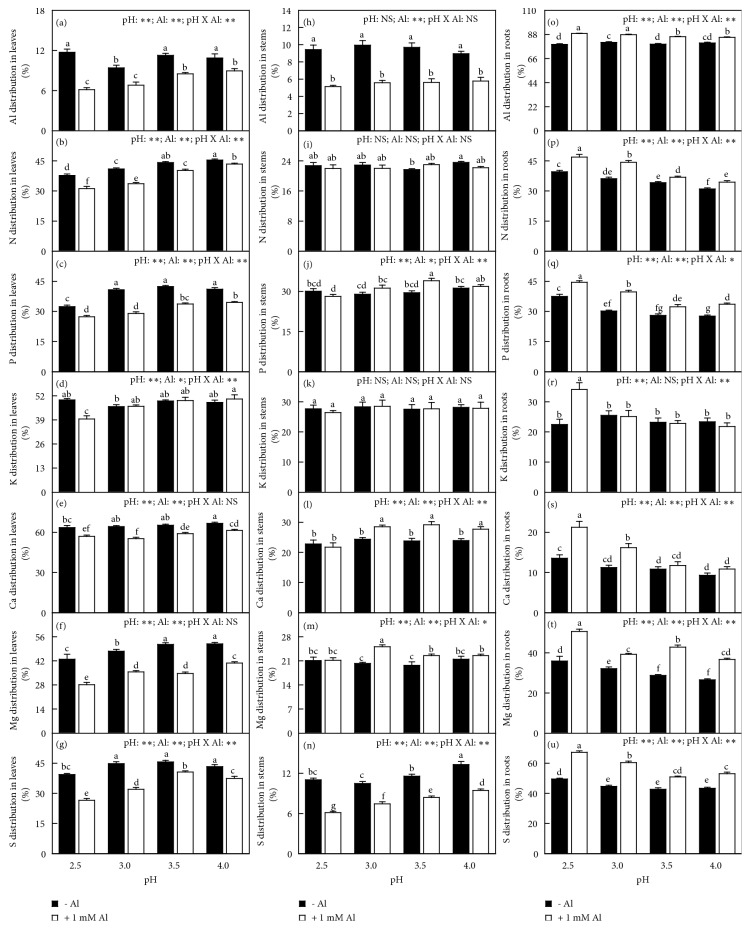
Effects of pH and Al interactions on (a, h, o) Al, (b, i, p) N, (c, j, q) P, (d, k, r) K, (e, l, s) Ca, (f, m, t) Mg, and (g, n, u) S distributions in* C. sinensis *(a-g) leaves, (h-n) stems, and (o-u) roots. Bars represent means ± SE (*n* = 8 except for 4 for K and Mg). Different letters above the bars indicate a significant difference at* P *< 0.05. NS, *∗* and *∗∗* indicate nonsignificant and significant at 5% and 1% level, respectively.

**Figure 9:**
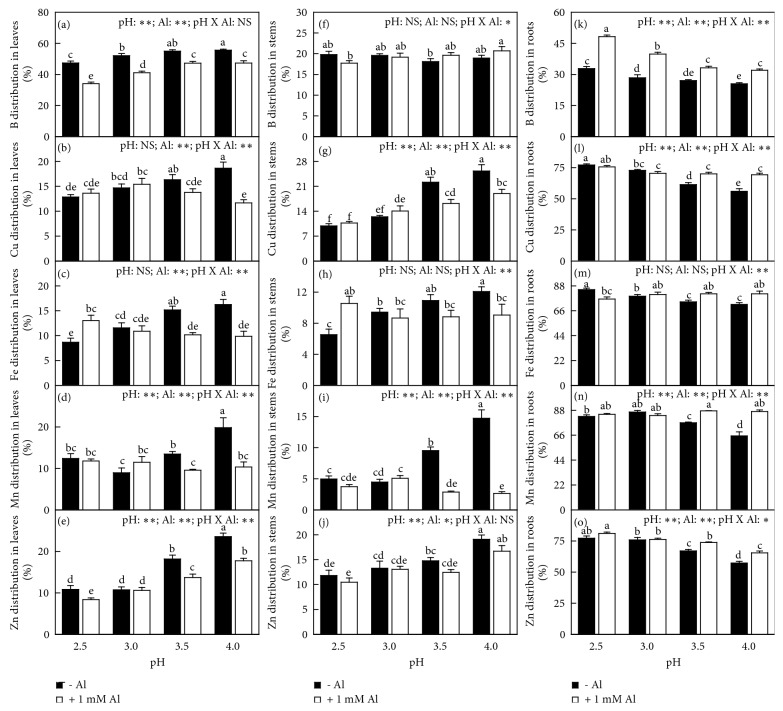
Effects of pH and Al interactions on (a, f, k) B, (b, g, l) Cu, (c, h, m) Fe, (d, i, n) Mn, and (e, j, o) Zn distribution in* C. sinensis *(a-e) leaves, (f-j) stems, and (k-o) roots. Bars represent means ± SE (*n* = 8 except for 4 for Fe and Mn). Different letters above the bars indicate a significant difference at* P *< 0.05. NS, *∗* and *∗∗* indicate nonsignificant and significant at 5% and 1% level, respectively.

Generally speaking, all element distributions in leaves and stems (roots) decreased (increased) or did not alter with decreasing pH with or without Al-toxicity. Al decreased (increased) or did not affect Al, N, P, K, S, B, Cu, Mn, and Zn distributions in leaves and stems (roots) with the exceptions that P distribution in stems and Cu distribution in leaves were increased by Al at pH 3.5. Al decreased Ca and Mg distributions in leaves and increased or did alter their distributions in stems and roots. At pH 2.5, Al increased (decreased) Fe distribution in leaves and stems (roots), but at pH 3.0-4.0, it decreased (increased) or did not alter Fe distribution in leaves and stems (roots). Interactions between pH and Al on element distributions were significant for all element distributions in leaves, stems, and roots except for the distributions of Ca, Mg, and B in leaves and Al, N, K, and Zn in stems (Figures 8-9).

### 3.5. PCA Loading Plots

**Figure 10:**
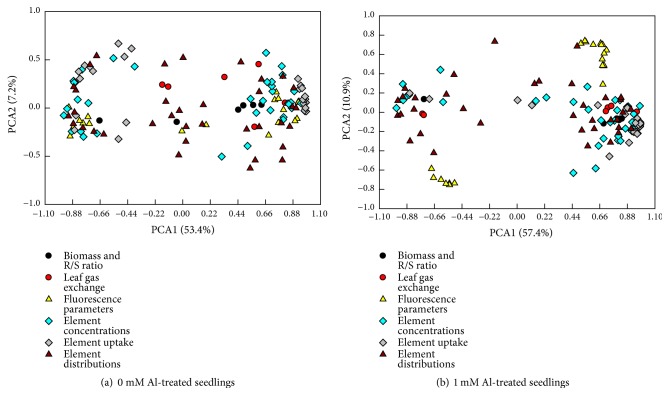
PCA loading plots of all physiological parameters for the (a) 0 and (b) 1 mM Al-treated* C. sinensis* seedlings submitted to pH 2.5, 3.0, 3.5, and 4.0.

PCA was carried out to examine the physiological patterns of* C. sinensis *seedlings in response to pH with or without Al-toxicity (Figure 10 and Tables [Supplementary-material supplementary-material-1]-[Supplementary-material supplementary-material-1]). The first two components contributed to 60.6% and 68.3% of the total variation in the 0 and 1 mM Al-treated* C. sinensis* seedlings, respectively. These parameters were more highly clustered in the 1 mM Al-treated seedlings than those in the 0 mM Al-treated ones. For the 0 mM Al-treated seedlings, PC1 was heavily loaded on N uptake per root DW (0.985), N uptake per plant (0.982), B uptake per root DW (0.980), B uptake per plant (0.977), Mg uptake per root DW (0.975), leaf N concentration (0.974), Ca uptake per root DW (0.972), Ca uptake per plant (0.963), K uptake per root DW (0.959), and P uptake per root DW (0.952) ([Supplementary-material supplementary-material-1]). For the 1 mM Al-treated seedlings, PC1 was the mostly affected by the alterations of stem N concentration (0.982), N uptake per root DW (0.981), Mg uptake per root DW (0.981), B uptake per root DW (0.980), Mg uptake per plant (0.978), B uptake per plant (0.977), N uptake per plant (0.977), Ca uptake per plant (0.967), Ca uptake per root DW (0.965), and leaf Mn concentration (-0.962) ([Supplementary-material supplementary-material-1]).

**Figure 11:**
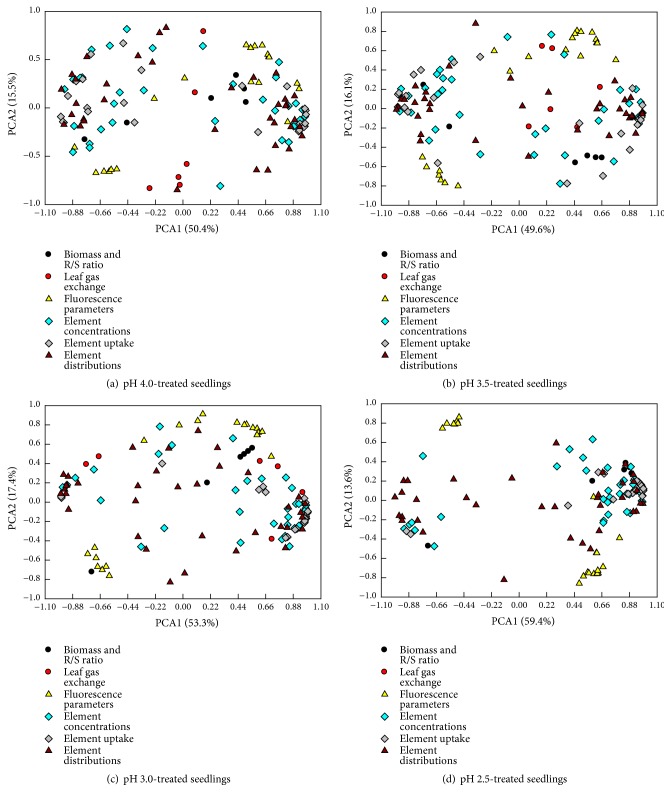
PCA loading plots of all physiological parameters for (a) pH 4.0-, (b) pH 3.5-, (c) pH 3.0-, and (d) pH 2.5-treated* C. sinensis* seedlings submitted to 0 and 1 mM Al.

Also, we determined the physiological patterns of* C. sinensis* seedlings in response to Al at pH 2.5, 3.0, 3.5, or 4.0 (Figure 11 and Tables [Supplementary-material supplementary-material-1]–[Supplementary-material supplementary-material-1]). The contribution of PC1 and PC2 to the total variation displayed little change as pH decreased from pH 4.0 to 3.5 and then increased with further decreasing pH. For the pH 4.0-treated seedlings, Ca uptake per root DW(0.981), Ca uptake per plant (0.979), K uptake per root DW (0.974), Al uptake per plant (-0.974), stem Mn concentration (0.973), Mg uptake per root DW (0.972), N uptake per root DW (0.970), Mg distribution in roots (-0.970), P uptake per root DW (0.965), and Al uptake per root DW (-0.958) contributed largely to PC1 ([Supplementary-material supplementary-material-1]). For the pH 3.5-treated seedlings, stem Mn concentration (0.985), N uptake per root DW (0.983), Mn distribution in stems (0.982), Mg distribution in leaves (0.982), Mn distribution in roots (-0.979), P uptake per root DW (0.977), leaf Mg concentration (0.974), root P concentration (0.970), Ca uptake per root DW (0.970), and Mn uptake per plant (-0.969) were the main contributors to PC1 ([Supplementary-material supplementary-material-1]). For the pH 3.0-treated seedlings, PC1 was mostly influenced by the alterations of Mg uptake per root DW (0.995), Mg uptake per plant (0.994), P uptake per root DW (0.982), leaf Mg concentration (0.982), P uptake per plant (0.982), N uptake per plant (0.980), N uptake per root DW(0.979), leaf P concentration (0.979), leaf Ca concentration (0.978), and S uptake per plant (0.978) ([Supplementary-material supplementary-material-1]). For the pH 2.5-treated seedlings, PC1 was largely accounted for by the modifications of S uptake per plant (0.991), N uptake per plant (0.991), S distribution in roots (-0.991), S uptake per root DW (0.990), N uptake per root DW(0.990), S distribution in leaves (0.987), Mg uptake per plant(0.985), leaf S concentration (0.984), Mg uptake per root DW(0.982), and K uptake per plant (0.980) ([Supplementary-material supplementary-material-1]).

## 4. Discussion

### 4.1. Interactive Effects of Al and Low pH on* C. sinensis* Seedlings Showed Synergism

The Al-induced alterations of most physiological parameters and OJIP transients became more pronounced with decreasing pH. Many parameters were altered by Al-toxicity only at pH 2.5-3.0, but unaffected at pH 3.5-4.0 (Figures 1–9 and [Supplementary-material supplementary-material-1] and [Supplementary-material supplementary-material-1]). The exception was that the Al-induced increase in leaf and stem level of B was greater at pH 3.5-4.0 than that at pH 2.5-3.0 or similar between the two (Figure 6). These findings indicated that the Al-induced alterations of these physiological parameters were intensified by low pH. Obviously, increasing the nutrient solution pH from 2.5 to 4.0 alleviated Al-toxicity of* C. sinensis* seedlings. This agrees with the results obtained on* Arabidopsis *[16], wheat [17], and* Eucalyptus *[18]. We observed that both the level of Al in roots, stems, and leaves and Al uptake per plant (root DW) increased with decreasing pH with or without Al-toxicity with the exception that Al uptake per plant was basically unchanged in response to pH in 1 mM Al-treated seedlings (Figures 5 and 7). The increased pH-induced decreases in the level of Al in roots, stems, and leaves and Al uptake per root DW might be responsible for the elevated pH-induced alleviation of* C. sinensis *Al-toxicity. Also, we observed that the low pH-induced alterations of most physiological parameters were greater in the 1 mM Al-treated seedlings than those in the 0 mM Al-treated ones (Figures 1–9 and [Supplementary-material supplementary-material-1] and [Supplementary-material supplementary-material-1]), demonstrating that the low pH-induced alterations of physiological parameters were enhanced by Al-toxicity. To conclude, there was a synergism between low pH and Al.

### 4.2. Al-Toxicity Increased Root Al Accumulation, Especially at Low pH

Plant Al-tolerance is associated not only with less uptake of Al by roots, but also with relatively less transport of Al from roots to shoots [29]. Previous studies indicated that the supply of S, B, and P decreased Al level in stems and leaves and increased or did not affect root Al level, thus alleviating* Citrus *Al-toxicity [6, 7, 23]. Thus, the Al-induced increase in root Al accumulation and decrease in leaf and stem Al accumulation (Figure 8) might be an adaptive strategy of* C. sinensis* to Al-toxicity. However, the increase in Al-tolerance due to the increased pH could not be explained in this way, because Al distribution in roots of the Al-treated seedlings was higher at pH 2.5-3.0 than that at pH 3.5-4.0. We found that increasing the nutrient solution pH from 2.5 to 4.0 decreased the level of Al in roots, stems, and leaves and the uptake of Al per root DW (Figures 5 and 7), which might play a key role in the increased pH-induced alleviation of* Citrus* Al-toxicity.

### 4.3. Increased Uptake and Levels of Nutrients Might Play a Role in the Elevated pH-Induced Alleviation of Al-Toxicity

Micromolar concentration of Al^3+^ can lead to a rapid inhibition of root growth and subsequently interfering with the uptake of nutrients [2, 20]. Al decreased the uptake of N, P, K, Ca, Mg, and Cu per plant at each given pH, especially at low pH with the exception that the uptake of Cu per plant was not altered by Al at pH 4.0. The uptake of S, B, Mn, and Zn per plant was increased and decreased by Al at pH 3.5-4 and pH 2.5-3, respectively (Figure 7). Regression analysis indicated that both whole plant DW and leaf CO_2_ assimilation decreased with decreasing uptake of N, P, K, Ca, Mg, S, B, or Fe per plant (Figures [Supplementary-material supplementary-material-1]-[Supplementary-material supplementary-material-1]). Generally viewed, Al decreased the levels of N, P, K, Ca, Mg, and S in roots, stems, and leaves, especially at pH 2.5-3.0 with the exceptions of a few, but increased the level of B in leaves, stems, and roots at each given pH. Also, B level in roots, stems, and leaves increased with increasing pH with or without Al-toxicity (Figures 5-6). Regression analysis showed that leaf CO_2_ assimilation decreased with decreasing leaf concentration of N, K, Ca, Mg, Fe, or Zn ([Supplementary-material supplementary-material-1]). The supply of Ca, Mg, S, B, and P can ameliorate Al-toxicity of plants [6, 7, 23, 30–35]. PCA showed that N, P, K, Ca, Mg, S, and B uptake per plant and/or per root DW was the main contributors to PC1 in the 0 mM Al-, 1 mM Al-, pH 4.0-, pH 3.5-, pH 3,0- and/or pH 2.5-treated seedlings (Tables [Supplementary-material supplementary-material-1]-[Supplementary-material supplementary-material-1]), demonstrating that the uptake of these elements might play a role in* Citrus* Al-toxicity (tolerance) and/or low pH-toxicity (tolerance). Thus, both the increased uptake per plant and/or root, stem, and leaf levels of N, P, Ca, K, Mg, S, and B might be involved in the increased pH-induced alleviation of* Citrus* Al-toxicity.

### 4.4. Causes for the Elevated pH-Induced Alleviation of Photosynthetic Decline in the Al-Treated Leaves

Although g_s_ in the Al-treated leaves increased with increasing pH (Figure 2(b)) and leaf CO_2_ assimilation increased with increasing g_s_ ([Supplementary-material supplementary-material-1]), the ameliorative action of the increased pH against the inhibitory effect of Al on photosynthesis (Figure 2(a)) could not explained by the increased g_s_ alone, because low pH increased or did not affect both C_i_ and C_i_/C_a_ ratio (Figure 2) and leaf CO_2_ assimilation decreased with increasing C_i_ or C_i_/C_a_ ratio ([Supplementary-material supplementary-material-1]).

Previous studies showed that the impaired whole photosynthetic electron transport chain from the donor side of PSII to the reduction of PSI end acceptors was the main cause contributing to the Al-induced inhibition of photosynthesis in* Citrus *leaves [27] and that the Al-induced impairment of the whole photosynthetic electron transport chain and the subsequent decline in leaf CO_2_ assimilation could be alleviated by the supply of B, S, and P [6, 7, 23]. We observed that Al-toxicity lowered F_v_/F_m_ (a good indicator of photoinhibitory effects on PSII) and *ψ*_Eo_ (ET_o_/TR_o_), increased DI_o_/RC, and altered greatly OJIP transients in low pH treated leaves (Figures 3-4), together demonstrating that photoinhibition occurred in these leaves [36, 37]. Increasing the nutrient solution pH from 2.5 to 4.0 prevented the Al-induced alterations of OJIP transients and all the 21 fluorescence parameters (Figures 3-4 and [Supplementary-material supplementary-material-1]). Regression analysis showed that there was a positive relationships between leaf CO_2_ assimilation and F_m_, F_v_, F_v_/F_o_, F_OEC_, maximum amplitude of IP phase, F_v_/F_m_, ET_o_/ABS, RE_o_/ABS, ET_o_/TR_o_, RE_o_/TR_o_, ET_o_/RC, RE_o_/RC, RE_o_/CS_o_, or PI_abs,total_, but a negative relationship between leaf CO_2_ assimilation and F_o_, M_o_, DI_o_/ABS, ABS/RC, DI_o_/RC, TR_o_/RC, or DI_o_/CS_o_ ([Supplementary-material supplementary-material-1]). These results suggested that the increased pH alleviated the Al-toxic impairment on the whole electron transport chain, thus preventing the Al-induced inhibition of photosynthesis.

Evidence shows that the deficiencies of mineral nutrients (N, P, K, Ca, and Mg) can impair the whole photosynthetic electron transport chain and cause a marked decline in leaf photosynthesis [38–43]. Here, the increased pH-induced alleviation of the Al-induced decreases in leaf levels of N, P, K, Ca, and Mg and their uptake per plant might be one of the causes for preventing the Al-induced decline in leaf CO_2_ assimilation, as indicated by the positive correlations between leaf CO_2_ assimilation and leaf level of N, K, Ca, or Mg and uptake per plant of N, P, K, Ca, or Mg ([Supplementary-material supplementary-material-1]). Previous study showed that Al-toxicity increased or did not affect B concentration in* Citrus grandis* roots, stem and leaves, but supply B alleviated the Al-induced impairment occurring in the whole photosynthetic electron transport chain and inhibition of photosynthesis [23]. Our results showed that B concentration in roots, stems, and leaves and its uptake increased with increasing pH (Figures 6-7) and that leaf CO_2_ assimilation increased with increasing B uptake per plant and displayed an increased trend with increasing leaf B concentration ([Supplementary-material supplementary-material-1]). These results indicated that the increased pH-induced increase in B uptake per plant might contribute to the alleviation of photosynthesis inhibition in Al-treated leaves. The antagonistic action of the increased pH against the inhibitory effect of Al-toxicity on leaf CO_2_ assimilation might also involve the increased pH-induced a decrease in leaf Al concentration (Figure 5(a)), as indicated by the negative and significant relationship between leaf CO_2_ assimilation and Al concentration ([Supplementary-material supplementary-material-1]) and the negative or positive relationships between leaf CO_2_ assimilation and Chl a fluorescence parameters ([Supplementary-material supplementary-material-1]). Based on these results, we concluded that increasing the solution pH from 2.5 to 4.0 mitigated the Al-induced impairment occurring on the whole photosynthetic electron transport chain, thus preventing the Al-induced decline in CO_2_ assimilation* via* decreasing the level of Al and increasing the uptake per plant of elements (N, P, K, Ca, Mg, and B) and their levels in leaves.

## 5. Conclusions

Our data clearly demonstrated that a synergism existed between low pH and Al and that increasing the nutrient solution pH from 2.5 to 4.0 alleviated the Al-toxicity of* C. sinensis *seedlings. Increasing pH decreased Al uptake per root DW and its level in roots, stems, and leaves and increased N, P, K, Ca, Mg, S, and B uptake per plant and their levels in roots, stems, and leaves. This might account for the increased pH-induced alleviation of* Citrus* Al-toxicity.

## Data Availability

The data used to support the findings of this study are available from the corresponding author upon request.

## Supplementary Materials

Supplementary Materials Figure S1: effects of pH and Al interactions on growth of* C. sinensis* seedlings. Figure S2: CO_2_ assimilation in relation to (a) stomatal conductance (g_s_), (b) intercellular CO_2_ concentration (C_i_), and (c) ratio of intercellular to ambient CO_2_ concentration (C_i_/C_a_) in* C. sinensis* leaves. Figure S3: effects of pH and Al interactions on the high-light-induced OJIP transients of dark-adapted* C. sinensis* leaves plotted on a logarithmic time scale (0.01 to 1000 ms). Figure S4: leaf CO_2_ assimilation in relation to 21 Chl a fluorescence parameters. Figure S5: element uptake per plant in relation to (a-l) whole plant DW and (m-x) root DW in* C. sinensis* seedlings. Figure S6: leaf CO_2_ assimilation in relation to (a-l) leaf element concentrations and (m-x) element uptake per plant in* C. sinensis* seedlings. Figure S7: leaf Al concentration in relation to 21 Chl a fluorescence parameters. Table S1: PCA for physiological parameters of 0 mM Al-treated* C. sinensis* seedlings. Table S2: PCA for physiological parameters of 1 mM Al-treated* C. sinensis* seedlings. Table S3: PCA for physiological parameters of pH 4-treated* C. sinensis* seedlings. Table S4: PCA for physiological parameters of pH 3.5-treated* C. sinensis* seedlings. Table S5: PCA for physiological parameters of pH 3-treated* C. sinensis* seedlings. Table S6: PCA for physiological parameters of pH 2.5-treated* C. sinensis* seedlings.
