# Supplementary material for: Increasing Nutrient Solution pH Alleviated Aluminum-Induced Inhibition of Growth and Impairment of Photosynthetic Electron Transport Chain in Citrus sinensis Seedlings
Source: Biomed Res Int. 2019 Aug 27;2019:9058715. doi: 10.1155/2019/9058715 (PMC6732596; doi:10.1155/2019/9058715)
Supplement: Supplementary Materials — Figure S1: effects of pH and Al interactions on growth of C. sinensis seedlings. Figure S2: CO2 assimilation in relation to (a) stomatal conductance (gs), (b) intercellular CO2 concentration (Ci), and (c) ratio of intercellular to ambient CO2 concentration (Ci/Ca) in C. sinensis leaves. Figure S3: effects of pH and Al interactions on the high-light-induced OJIP transients of dark-adapted C. sinensis leaves plotted on a logarithmic time scale (0.01 to 1000 ms). Figure S4: leaf CO2 assimilation in relation to 21 Chl a fluorescence parameters. Figure S5: element uptake per plant in relation to (a-l) whole plant DW and (m-x) root DW in C. sinensis seedlings. Figure S6: leaf CO2 assimilation in relation to (a-l) leaf element concentrations and (m-x) element uptake per plant in C. sinensis seedlings. Figure S7: leaf Al concentration in relation to 21 Chl a fluorescence parameters. Table S1: PCA for physiological parameters of 0 mM Al-treated C. sinensis seedlings. Table S2: PCA for physiological parameters of 1 mM Al-treated C. sinensis seedlings. Table S3: PCA for physiological parameters of pH 4-treated C. sinensis seedlings. Table S4: PCA for physiological parameters of pH 3.5-treated C. sinensis seedlings. Table S5: PCA for physiological parameters of pH 3-treated C. sinensis seedlings. Table S6: PCA for physiological parameters of pH 2.5-treated C. sinensis seedlings. [file 9058715.f1.doc]

# BioMed Research International

# Increasing Nutrient Solution pH Alleviated Aluminum-induced Inhibition of Growth and Impairment of Photosynthetic Electron Transport Chain in *Citrus sinensis* Seedlings

Tao-Yu Yang,^1^ Li-Ya Cai,^1^ Yi-Ping Qi,^2^ Lin-Tong Yang,^1^ Ning-Wei Lai,^1^ and Li-Song Chen,^1,3^

*^1^ Institute of Plant Nutritional Physiology and Molecular Biology, College of Resources and Environment, Fujian Agriculture and Forestry University, Fuzhou 350002, China.*
^2^*Institute of Materia Medica, Fujian Academy of Medical Sciences, Fuzhou 350002, China.*

^3^*Fujian Provincial Key Laboratory of Soil Environmental Health and Regulation, College of Resources and Environment, Fujian Agriculture and Forestry University, Fuzhou 350002, China.*

Correspondence should be addressed to Li-Song Chen; lisongchen2002@hotmail.com, lisongchen@fafu.edu.cn

## Abstract

Although the physiological and molecular responses of *Citrus* to Al-toxicity or low pH have been examined in some details, little information is available on *Citrus* responses to pH and aluminum (Al) interactions. *Citrus sinensis* seedlings were irrigated for 18 weeks with nutrient solution at a concentration of 0 or 1 mM AlCl_3_•6H_2_O and a pH of 2.5, 3.0, 3.5 or 4.0. Thereafter, biomass, root, stem and leaf concentrations of Al and nutrients, leaf gas exchange, chlorophyll a fluorescence (OJIP) transients and related parameters were investigated to understand the physiological mechanisms underlying the elevated pH-induced alleviation of *Citrus* toxicity. Increasing the nutrient solution pH from 2.5 to 4.0 alleviated the Al-toxic effects on biomass, photosynthesis, OJIP transients and related parameters, and element concentrations, uptake and distributions. In addition, low pH effects on the above physiological parameters were intensified by Al-toxicity. Evidently, a synergism existed between low pH and Al-toxicity. Increasing pH decreased Al uptake per root dry weight and its concentration in roots, stems and leaves, and increased nitrogen, phosphorus, calcium, magnesium, sulfur and boron uptake per plant and their concentrations in roots, stems and leaves. This might be responsible for the elevated pH-induced alleviation of growth inhibition and the impairment of the whole photosynthetic electron transport chain, thus preventing the decrease of CO_2_ assimilation.

## Introduction

Aluminum (Al) exists mostly as silicate or oxide precipitates that are biologically inactive in neutral or moderately acidic soils. However, Al solubility increases greatly in acidic soils (pH < 5), resulting in the release of phytotoxic Al^3+^ from clay minerals into soil solution [1]. Micromolar concentration of Al^3+^ can cause a rapid inhibition of root elongation and subsequently impair the uptake of water and nutrients, leading to poor growth and yield loss of crops [2]. Therefore, Al-toxicity in acid soils has been regarded as a major factor limiting crop productivity worldwide, since ~ 30% of free ice land is acidic [3]. Furthermore, soil acidity is becoming an increasingly serious problem due to the improper farming practices and environmental deterioration [4]. In recent decades, many researchers have investigated Al-toxic effects on plant growth [5-6], uptake of nutrients [6-7], leaf CO_2_ assimilation [8-11] and photosynthetic electron transport [12-13].

The toxicity of Al to plants depends not only on their Al-tolerance, but also on the soil properties, primarily pH [14]. Hence, it is necessary to investigate the combination effects of Al and pH on plants to better understand the adaption of plants to acidic soils with high active Al. However, such data are very rare, because Al-toxicity and low pH are almost examined separately in different experiments. To our best knowledge, most of studies regarding pH effects on plant Al-toxicity have focused on the pH-induced alterations in toxic Al species and activities in solution, root growth, and root tissue (cell sap) Al concentration, and the results are not consistent [1, 14-15]. Because Al-toxicity occurs mainly on acidic soils, it is generally believed that the lower the pH in the culture medium, the greater the toxicity of Al to plants. Degenhardt et al. [16] reported that an Al-induced increase in rhizosphere pH was responsible for the Al-resistance in the *Arabidospsis alr*-104 mutant, and that increasing the solution pH from 4.4 to 4.5 improved root growth rate of both *alr*-104 mutant and wild type under Al-toxicity. Wang et al. [17] observed that the Al-tolerant wheat cultivar had higher capacity to keep higher rhizosphere pH relative to the Al-sensitive, and that increasing the solution pH from 4.5 to 5.0 enhanced the Al-resistance of wheat. Using *Eucalyptus* trees, Yang et al. [18] found that raising the nutrient solution pH from 3.0 to 4.0 increased net photosynthesis and transpiration under Al-toxicity. However, Kinraide [19] found that the activities of Al^3+^ in soil solutions peaked at ~ pH 4.1 in the range of pH 3.5 to 5.5, implying that Al-toxicity in soils with pH 4.1 might be more severe than that in more acidic or alkaline soils. Al level in rice root cell sap increased as solution pH increased from 4.0 to 6.0 [20].

*Citrus* often display poor growth and a shortened lifetime in low pH soils with high active Al [21]. In China, *Citrus* are cultivated mainly on acidic soils. Moreover, soil acidity is becoming an increasing urgent problem in some *Citrus* orchards [22]. Although the physiological and molecular responses of *Citrus* to Al-toxicity or low pH have been investigated in some details [9, 23-25], very scarcity is known on *Citrus* responses to pH and Al interactions. Preliminary study showed that increasing nutrient solution pH prevented Al-toxic effects on *Citrus* growth and photosynthesis. Here, we used *C. sinensis* seedlings as materials and investigated the effects of pH and Al interactions on biomass; Al and nutrient elements in roots, stems and leaves; gas exchange, chlorophyll (Chl) a fluorescence (OJIP) transients and related parameters in leaves. Our objective was to determine the physiological mechanisms underlying the increased pH-induced alleviation of *Citrus* Al-toxicity.

## Materials and Methods

### *2.1. Culture and Treatments of Seedlings.*Seedling culture and treatments were conducted according to Guo et al. [26] with some modifications. Five-week-old ‘Xuegan’ (*C. sinensis*) seedlings were transplanted to 6L pots (two per pot) containing sand thoroughly washed with tap water, and then planted in a greenhouse under a natural photoperiod at Fujian Agriculture and Forestry University, Fuzhou with an annual average relative humidity, temperature and sunlight of ~ 76%, 20 °C and 16 h, respectively. Six weeks after transplanting, each pot was replenished every day with freshly prepared nutrient solution [i.e., macronutrients (in mM): Ca(NO_3_)_2_,1; KNO_3_, 1; MgSO_4_, 0.5; KH_2_PO_4_, 0.1; and micronutrients (in μM): Fe-EDTA, 20; ZnSO_4_, 2; MnCl_2_, 2; CuSO_4_, 0.5; and (NH_4_)_6_Mo_7_O_24_, 0.065] at a concentration of 0 or 1 mM AlCl_3_•6H_2_O and a pH of 2.5, 3.0, 3.5 or 4.0 for 18 weeks until a portion of nutrient solution began to leak out from a hole at the bottom of the pot (~ 500 mL). The pH of the solution was adjusted by NaOH or HCl before irrigation. At the end of the experiment, fully expanded (~ 7-week-old) leaves were used for the assays of all physiological parameters.

### *2.2. Measurements of Root, Stem and Leaf Dry Weights*. At the end of the experiment, ten seedlings per treatment from different pots were harvested, and then divided into roots, stems and leaves. Their dry weight (DW) were weighted after being dried to a constant weight at 70 °C.

### *2.3. Measurements of Leaf OJIP Transients and Calculation of Related Parameters.* Leaf OJIP transients were measured in 3 h dark-adapted seedlings at room temperature using a Handy Plant Efficiency Analyzer (Handy PEA, Hansatech Instruments Limited, Norfolk, UK). All fluorescence parameters were calculated according to Jiang et al. [27] and Banks [28].

### *2.4. Leaf Gas Exchange.* Leaf gas exchange was measured with a CIRAS-2 portable photosynthesis system (PP systems, Herts, UK) at a leaf temperature of ~ 26 °C, a controlled light intensity of ~ 1000 μmol m^-2^ s^-1^ and a controlled CO_2_ concentration of ~ 380 μmol mol^-1^ between 9 and 12 a.m. on a sunny day. The flow rate through the 2.5 cm^2^ leaf chamber was kept at 200 μmol s^-1^. Water use efficiency (WUE) was calculated as: CO_2_ assimilation/transpiration rate.

### *2.5. Assays of Elements, and Calculation of Element Uptake and Distributions in Roots, Stems and Leaves*. The small first- and second-order fibrous roots (< 2.0 mm diameter), the middle sections of stems, and the recent fully expanded mature (~ 7-week-old) leaves were used for the measurements of elements [26]. Al was assayed with a NexION 300X Inductively Coupled Plasma Mass Spectrometer (ICP-MS, PerkinElmer, CT, USA). Phosphorus (P) was measured colorimetrically as blue molybdate-phosphate complexes. Potassium (K) was assayed with a FP640 Flame Photometry (Shanghai Precision Scientific Instrument Co., Ltd, Shanghai, China). Calcium (Ca), magnesium (Mg), iron (Fe), manganese (Mn), copper (Cu) and zinc (Zn) were determined with a PinAAcle 900F Atomic Absorption Spectrometer (Perkinelmer Singapore Pte Ltd, Singapore). Sulfur (S) was determined by the simple turbidimetric method. Nitrogen (N) was assayed by the Kjeldahl method with a Kjeltec 8200 Auto Distillation (FOSS Analytical AB, Höganäs, Sweden). Boron (B) was determined by the curcumin method [22, 24].

Element uptake and distributions were calculated as described previously [26].

### *2.6. Data Analysis*. There were 12 pots (24 seedlings) per treatment in a completely randomized design. Results represented the mean ± SE of 4 - 12 replicates (one plant from different pot per treatment) except for the mean OJIP transients and the different expressions derived from the mean transients. Significant differences among the eight treatments were analysed by four (pH values) × two (Al levels) ANOVA and followed by the least significant difference (LSD) at *P*< 0.05 level.

Principal component analysis (PCA) was performed using a SPSS® statistical software (version 17.0, IBM, NY, USA) [26].

## 3. Results

### *3.1. Seedling Growth*. Without Al-toxicity, only pH 2.5 decreased stem, leaf, shoot and whole plant DW and increased root DW/shoot DW ratio relative to control (pH 4.0); with Al-toxicity, all the six parameters kept stable when pH decreased from 4.0 to 3.5, then root, stem, leaf, shoot and whole plant DW decreased and root DW/shoot DW ratio increased with further decreasing pH. Al decreased or did not alter stem, leaf, shoot and whole plant DW, but it increased or did not affect root DW and root DW/shoot DW ratio. The interactive effects of pH and Al were significant for root DW, whole plant DW and root DW/shoot DW ratio. Many rotten and died fibrous roots were observed in the pH 2.5 + 1 mM Al-treated seedlings (Figure 1 and Figure S1).

### Figure 1: Effects of pH and Al interactions on (a) root, (b) stem, (c) leaf, (d) shoot and (e) whole plant DW, and (f) root DW/shoot DW ratio in *C. sinensis* seedlings. Bars represent means ± SE (*n* = 10). Different letters above the bars indicate a significant difference at *P* < 0.05. NS, * and ** indicate nonsignificant, significant at 5% and 1% level, respectively.

### *3.2. Leaf Gas Exchange.* Without Al-toxicity, leaf stomatal conductance (g_s_) and transpiration rate (CO_2_ assimilation) did not change as pH decreased from 4.0 to 3.0 (3.5), then decreased with further decreasing pH, but intercellular CO_2_ concentration (C_i_), the ratio of intercellular to ambient CO_2_ concentration (C_i_/C_a_) and WUE did not alter in response to pH. With Al-toxicity, leaf CO_2_ assimilation, g_s_, transpiration rate and WUE kept unchanged as pH decreased from 4.0 to 3.5, then decreased with further decreasing pH, but both C_i_ and C_i_/C_a_ ratio were the highest at pH 2.5. Interactions between pH and Al on leaf gas exchange were significant only for CO_2_ assimilation (Figure 2).

Regression analysis showed that CO_2_ assimilation increased with increasing g_s_, but it increased with decreasing C_i_ and C_i_/C_a_ ratio in leaves (Figure S2).

### Figure 2: Effects of pH and Al interactions on (a) CO_2_ assimilation, (b) stomatal conductance (g_s_), (c) intercellular CO_2_ concentration (C_i_), (d) ratio of intercellular to ambient CO_2_ concentration (C_i_/C_a_), (e) transpiration rate and (f) WUE in *C. sinensis* leaves. Bars represent means ± SE (*n* = 5). Different letters above the bars indicate a significant difference at *P* < 0.05. NS, * and ** indicate nonsignificant, significant at 5% and 1% level, respectively.

*3.3. Leaf OJIP Transients and Related Parameters.* OJIP transients from the 0 mM Al-treated leaves displayed little alterations in response to pH except for a slight increase in O-step at pH 2.5 compared with OJIP transients from the pH 4.0 + 0 mM Al-treated leaves. Al increased the heterogeneity of samples, especially at pH 2.5. OJIP transients from the 1 mM Al-treated leaves displayed an increased O-step at pH 2.5-3.0 and a suppressed P-step at pH 2.5 compared with OJIP transients from the pH 4.0 + 1 mM Al-treated leaves (Figure S3). OJIP transients from the low pH and/or Al-treated leaves had positive ΔL-, ΔK-, ΔJ- and ΔI-bands around 150 μs, 300 μs, 2 ms and 30 ms compared with OJIP transients from the pH 4.0 + 0 mM Al-treated leaves, respectively. The positive ΔL-, ΔK-, ΔJ- and ΔI-bands were the most pronounced from the leaves treated by pH 2.5 + 1 mM Al (Figure 3).

### Figure 3: Effects of pH and Al interactions on the mean OJIP transients expressed as the kinetics of relative variable fluorescence: (a) between F_o_ and F_m_: V_t_ = (F_t_ – F_o_)/(F_m_ – F_o_) and (b) the differences of the eight samples to the reference sample submitted to pH 4.0 + 0 mM Al (ΔV_t_); (c) between F_o_ and F_300μs_: W_K_ = (F_t_ – F_o_)/(F_300μs_ – F_o_) and (d) the differences of the six samples to the reference sample submitted to pH 4.0 + 0 mM Al (ΔW_K_).

Without Al-toxicity, minimum fluorescence (F_o_), maximum fluorescence (F_m_), maximum variable fluorescence (F_v_), maximum primary yield of photochemistry of photosystem II (PSII; F_v_/F_o_), fraction of oxygen evolving complex (OEC) relative to control (F_OEC_), maximum amplitude of IP phase, maximum PSII efficiency of dark-adapted leaves (F_v_/F_m_), quantum yield for energy dissipation (DI_o_/ABS), quantum yield for electron transport (ET_o_/ABS), quantum yield for the reduction of end acceptors of PSI per photon absorbed (RE_o_/ABS), efficiency with which a trapped exciton can move an electron into the electron transport chain from Q_A_^-^ to the PSI end electron acceptors (RE_o_/TR_o_), absorption flux per reaction centre (RC, ABS/RC), dissipated energy flux per RC (DI_o_/RC), electron transport flux per RC (ET_o_/RC), reduction of end acceptors at PSI electron acceptor side (RE_o_/RC), dissipated energy flux per cross section (CS, DI_o_/CS_o_), reduction of end acceptors at PSI electron acceptor side (RE_o_/CS_o_) and total performance index (PI_abs,total_) displayed little change in response to pH except for increased F_o_ and TR_o_/RC, and decreased F_v_/F_o_, F_OEC_, maximum amplitude of IP phase, ET_o_/ABS, RE_o_/ABS, RE_o_/TR_o_, ET_o_/RC, and PI_abs,total_ at pH 2.5. However, approximated initial slope (in ms^-1^) of the fluorescence transient V = f(t) (M_o_) and trapped energy flux per RC (TR_o_/RC) increased, and probability that a trapped exciton moves an electron into the electron transport chain beyond Q_A_^-^ (ψ_Eo_ or ET_o_/TR_o_) decreased with decreasing pH (Figure 4).

### Figure 4: Effects of pH and Al interactions on 21 Chl a fluorescence parameters in dark-adapted *C. sinensis* leaves. Bars represent means ± SE (*n* = 12). Different letters above the bars indicate a significant difference at *P* < 0.05. NS, * and ** indicate nonsignificant, significant at 5% and 1% level, respectively.

With Al-toxicity, DI_o_/ABS, ABS/RC, DI_o_/RC and DI_o_/CS_o_ (F_o_, M_o_ and TR_o_/RC) did not change as pH decreased from 4.0 to 3.0 (3.5), and then increased with further decreasing pH. By contrast, F_v_/F_o_, F_OEC_, maximum amplitude of IP phase, ET_o_/ABS, RE_o_/ABS, ET_o_/TR_o_, RE_o_/TR_o_, RE_o_/RC, RE_o_/CS_o_ and PI_abs,total_ (F_m_, F_v_, F_v_/F_m_ and ET_o_/RC) did not change as pH decreased from 4.0 to 3.5 (3.0), and then decreased with further decreasing pH (Figure 4).

Al-toxicity increased or did not affect F_o_, M_o_, DI_o_/ABS, ABS/RC, DI_o_/RC, TR_o_/RC, and DI_o_/CS_o_, but decreased or did not alter the other fluorescence parameters. Interactions between pH and Al on Chl a fluorescence parameters were significant only for F_o_, F_m_, F_v_, F_v_/F_o_, F_v_/F_m_, DI_o_/ABS, ET_o_/ABS, RE_o_/ABS, ABS/RC, DI_o_/RC, RE_o_/RC and DI_o_/CS_o_ (Figure 4).

Leaf CO_2_ assimilation increased with increasing F_m_, F_v_, F_v_/F_o_, F_OEC_, maximum amplitude of IP phase, F_v_/F_m_, ET_o_/ABS, RE_o_/ABS, ET_o_/TR_o_, RE_o_/TR_o_, ET_o_/RC, RE_o_/RC, RE_o_/CS_o_ or PI_abs,total_, respectively, but it decreased with increasing F_o_, M_o_, DI_o_/ABS, ABS/RC, DI_o_/RC, TR_o_/RC or DI_o_/CS_o_, respectively (Figure S4).

### *3.4. Concentrations, Uptake and Distributions of Elements*. Without Al-toxicity, the levels of Al, S and Mn in leaves, stems and roots, Cu in leaves and roots, Zn in stems and leaves, and Fe in roots increased or did not change with decreasing pH, but the levels of P, N, Ca, K, Mg and B in leaves, stems and roots, Fe in leaves and stems, Cu in stems, and Zn in leaves decreased or did not alter with decreasing pH (Figures 5-6).

With Al-toxicity, the levels of Al and Mn in leaves, stems and roots, and Cu in leaves increased or did not change with decreasing pH. The levels of N, P, K, Ca, Mg, B and Fe in leaves, stems and roots, Cu in stems and roots, Zn in leaves and stems, and S in stems decreased or did not change with decreasing pH. The levels of S in leaves and Zn in roots (S in roots) increased as pH decreased from 4.0 to 3.5 (3.0), and then decreased with further decreasing pH (Figures 5-6).

Al-toxicity increased or did not alter the levels of Al and B in leaves, stems and roots, Zn in leaves and stems, Mn in leaves, and S in roots at each given pH with the exception that the levels of Zn in leaves and S in roots were decreased by Al at pH 2.5, but decreased or did not alter the levels of N, P, K, Ca, Mg, Cu and Fe in leaves, stems and roots, S and Mn in stems, and Zn in roots with the exception that root Fe level was increased by Al at pH 4.0. The levels of S in leaves and Mn in roots were decreased by Al at pH 2.5-3.0, but increased or unaffected by Al at pH 3.5-4.0. Interactions between pH and Al on element levels were significant only for N, S, Mn and Zn levels in leaves, stems and roots, K level in leaves and roots, P and Ca levels in leaves, B level in stems, Mg, Cu and Fe levels in roots (Figures 5-6).

### Figure 5: Effects of pH and Al interactions on concentrations of (a, h, o) Al, (b, i, p) N, (c, j, q) P, (d, k, r) K, (e, l, s) Ca, (f, m, t) Mg and (g, n, u) S in *C. sinensis* (a-g) leaves, (h-n) stems and (o-u) roots. Bars represent means ± SE (*n* = 8 except for 4 for K and Mg). Different letters above the bars indicate a significant difference at *P* < 0.05. NS, * and ** indicate nonsignificant, significant at 5% and 1% level, respectively.

### Figure 6: Effects of pH and Al interactions on concentrations of (a, f, k) B, (b, g, l) Cu, (c, h, m) Fe, (d, i, n) Mn and (e, j, o) Zn in *C. sinensis* (a-e) leaves, (f-j) stems and (k-o) roots. Bars represent means ± SE (*n* = 8 except for 4 for Fe and Mn). Different letters above the bars indicate a significant difference at *P* < 0.05. NS, * and ** indicate nonsignificant, significant at 5% and 1% level, respectively.

Without Al-toxicity, the uptake of Al, S, Cu, Fe, Mn and Zn per plant (root DW) increased or did not alter with decreasing pH with the exception that the uptake of Mn and Zn per plant was higher at pH 3.0 than that at pH 2.5, but the uptake of the other elements per plant (root DW) decreased or kept unchanged with decreasing pH. With Al-toxicity, the uptake of Al and Mn per plant (root DW) increased or did not alter with decreasing pH with the exception that the uptake of Mn per plant was slightly higher at pH 3.5 than that at pH 2.5, but the uptake of the other elements per plant (root DW) decreased or kept unchanged with decreasing pH with the exception that the uptake of S per plant (root DW) was higher at pH 3.5 than that at pH 4.0. Al-toxicity increased the uptake of Al per plant (root DW), and increased or did not alter the uptake of B per plant (root DW) with the exception that the uptake of B per plant was decreased by Al at pH 2.5, but it decreased the uptake of N, P, K, Ca and Mg per plant (root DW), and decreased or did not change the uptake of S, Cu, Fe and Zn per root DW. The uptake of S, Cu, Fe, Mn and Zn per plant and of Mn per root DW was decreased by Al at pH 2.5-3.0, but increased or unaltered by Al at pH 3.5-4.0. Interactions between pH and Al on the uptake of elements were significant for the uptake of all elements except for the uptake of Al, N and K per root DW (Figure 7).

### Figure 7: Effects of pH and Al interactions on element uptake (a-l) per plant and (m-x) per root DW in *C. sinensis* seedlings. Bars represent means ± SE (*n* = 8 except for 4 for K, Mg, Fe and Mn). Different letters above the bars indicate a significant difference at *P* < 0.05. NS, * and ** indicate nonsignificant, significant at 5% and 1% level, respectively.

Whole plant (root) DW increased with increasing uptake of N, P, K, Ca, Mg, S, B or Fe (S, B or Fe) per plant (Figure S5).

Leaf CO_2_ assimilation decreased with increasing leaf Al or Mn concentration, but it increased with increasing leaf N, K, Ca, Mg, Fe or Zn concentrations. Leaf CO_2_ assimilation increased with increasing uptake of N, P, K, Ca, Mg, S, B or Fe per plant (Figure S6).

Leaf Al increased with increasing F_o_, M_o_, DI_o_/ABS, ABS/RC, DI_o_/RC, TR_o_/RC or DI_o_/CS_o_, respectively, but it decreased with increasing F_m_, F_v_, F_v_/F_o_, F_OEC_, maximum amplitude of IP phase, F_v_/F_m_, ET_o_/ABS, RE_o_/ABS, ET_o_/TR_o_, RE_o_/TR_o_, ET_o_/RC, RE_o_/RC, RE_o_/CS_o_ or PI_abs,total_, respectively (Figure S7).

Generally speaking, all element distributions in leaves and stems (roots) decreased (increased) or did not alter with decreasing pH with or without Al-toxicity. Al decreased (increased) or did not affect Al, N, P, K, S, B, Cu, Mn and Zn distributions in leaves and stems (roots) with the exceptions that P distribution in stems and Cu distribution in leaves were increased by Al at pH 3.5. Al decreased Ca and Mg distributions in leaves and increased or did alter their distributions in stems and roots. At pH 2.5, Al increased (decreased) Fe distribution in leaves and stems (roots), but at pH 3.0-4.0, it decreased (increased) or did not alter Fe distribution in leaves and stems (roots). Interactions between pH and Al on element distributions were significant for all element distributions in leaves, stems and roots except for the distributions of K, Mg and B in leaves, and Al, N, K and Zn in stems (Figures 8-9).

### Figure 8: Effects of pH and Al interactions on (a, h, o) Al, (b, i, p) N, (c, j, q) P, (d, k, r) K, (e, l, s) Ca, (f, m, t) Mg and (g, n, u) S distributions in *C. sinensis* (a-g) leaves, (h-n) stems and (o-u) roots. Bars represent means ± SE (*n* = 8 except for 4 for K and Mg). Different letters above the bars indicate a significant difference at *P* < 0.05. NS, * and ** indicate nonsignificant, significant at 5% and 1% level, respectively.

### Figure 9: Effects of pH and Al interactions on (a, f, k) B, (b, g, l) Cu, (c, h, m) Fe, (d, i, n) Mn and (e, j, o) Zn distribution in *C. sinensis* (a-e) leaves, (f-j) stems and (k-o) roots. Bars represent means ± SE (*n* = 8 except for 4 for Fe and Mn). Different letters above the bars indicate a significant difference at *P* < 0.05. NS, * and ** indicate nonsignificant, significant at 5% and 1% level, respectively.

### *3.5. PCA Loading Plots*. PCA was carried out to examine the physiological patterns of *C. sinensis* seedlings in response to pH with or without Al-toxicity (Figure 10 and Tables S1-S2). The first two components contributed to 62.0% and 68.4% of the total variation in the 0 and 1 mM Al-treated *C. sinensis* seedlings, respectively. These parameters were more highly clustered in the 1 mM Al-treated seedlings than those in the 0 mM Al-treated ones. For the 0 mM Al-treated seedlings, PC1 was heavily loaded on N uptake per root DW (0.985), N uptake per plant (0.982), B uptake per root DW (0.980), B uptake per plant (0.977), Mg uptake per root DW (0.975), leaf N concentration (0.974), Ca uptake per root DW (0.972), Ca uptake per plant (0.963), K uptake per root DW (0.959) and P uptake per root DW (0.952) (Table S1). For the 1 mM Al-treated seedlings, PC1 was the mostly affected by the alterations of stem N concentration (0.982), N uptake per root DW (0.981), Mg uptake per root DW (0.981), B uptake per root DW (0.980), Mg uptake per plant (0.978), B uptake per plant (0.977), N uptake per plant (0.977), Ca uptake per plant (0.967), Ca uptake per root DW (0.965) and leaf Mn concentration (-0.962) (Table S2).

###

### Figure 10: PCA loading plots of all physiological parameters for the (a) 0 and (b) 1 mM Al-treated *C. sinensis* seedlings submitted to pH 2.5, 3.0, 3.5 and 4.0.

Also, we determined the physiological patterns of *C. sinensis* seedlings in response to Al at pH 2.5, 3.0, 3.5 or 4.0 (Figure 11 and Tables S3-S6). The contribution of PC1 and PC2 to the total variation displayed little change as pH decreased from pH 4.0 to 3.5, then increased with further decreasing pH. For the pH 4.0-treated seedlings, Ca uptake per root DW(0.981), Ca uptake per plant (0.979), K uptake per root DW (0.974), Al uptake per plant (-0.974), stem Mn concentration (0.973), Mg uptake per root DW (0.972), N uptake per root DW (0.970), Mg distribution in roots (-0.970), P uptake per root DW (0.965) and Al uptake per root DW (-0.958) contributed largely to PC1 (Table S3). For the pH 3.5-treated seedlings, stem Mn concentration (0.985), N uptake per root DW (0.983), Mn distribution in stems (0.982), Mg distribution in leaves (0.982), Mn distribution in roots (-0.979), P uptake per root DW (0.977), leaf Mg concentration (0.974), root P concentration (0.970), Ca uptake per root DW (0.970) and Mn uptake per plant (-0.969) were the main contributors to PC1 (Table S4). For the pH 3.0-treated seedlings, PC1 was mostly influenced by the alterations of Mg uptake per root DW (0.995), Mg uptake per plant (0.994), P uptake per root DW (0.982), leaf Mg concentration (0.982), P uptake per plant (0.982), N uptake per plant (0.980), N uptake per root DW(0.979), leaf P concentration (0.979), leaf Ca concentration (0.978) and S uptake per plant (0.978) (Table S5). For the pH 2.5-treated seedlings, PC1 was largely accounted for by the modifications of S uptake per plant (0.991), N uptake per plant (0.991), S distribution in roots (-0.991), S uptake per root DW (0.990), N uptake per root DW(0.990), S distribution in leaves (0.987), Mg uptake per plant(0.985), leaf S concentration (0.984), Mg uptake per root DW(0.982) and K uptake per plant (0.980) (Table S6).

### Figure 11: PCA loading plots of all physiological parameters for (a) pH 4.0-, (b) pH 3.5-, (c) pH 3.0- and (d) pH 2.5-treated *C. sinensis* seedlings submitted to 0 and 1 mM Al.

## 4. Discussion

### *4.1. Interactive Effects of Al and Low pH on C. sinensis Seedlings Showed Synergism*. The Al-induced alterations of most physiological parameters and OJIP transients became more pronounced with decreasing pH. Many parameters were altered by Al-toxicity only at pH 2.5-3.0, but unaffected at pH 3.5-4.0 (Figures 1-9, S1 and S3). The exception was that the Al-induced increase in leaf and stem level of B was greater at pH 3.5-4.0 than that at pH 2.5-3.0 or similar between the two (Figure 6). These findings indicated that the Al-induced alterations of these physiological parameters were intensified by low pH. Obviously, increasing the nutrient solution pH from 2.5 to 4.0 alleviated Al-toxicity of *C. sinensis* seedlings. This agrees with the results obtained on *Arabidopsis* [16], wheat [17] and *Eucalyptus* [18]. We observed that both the level of Al in roots, stems and leaves and Al uptake per plant (root DW) increased with decreasing pH with or without Al-toxicity with the exception that Al uptake per plant was basically unchanged in response to pH in 1 mM Al-treated seedlings (Figures 5, 7). The increased pH-induced decreases in the level of Al in roots, stems and leaves and Al uptake per root DW might be responsible for the elevated pH-induced alleviation of *C. sinensis* Al-toxicity. Also, we observed that the low pH-induced alterations of most physiological parameters were greater in the 1 mM Al-treated seedlings than those in the 0 mM Al-treated ones (Figures 1-9, S1 and S3), demonstrating that the low pH-induced alterations of physiological parameters were enhanced by Al-toxicity. To conclude, there was a synergism between low pH and Al.

### *4.2. Al-toxicity Increased Root Al Accumulation, Especially at Low pH*. Plant Al-tolerance is associated not only with less uptake of Al by roots, but also with relatively less transport of Al from roots to shoots [29]. Previous studies indicated that the supply of S, B and P decreased Al level in stems and leaves and increased or did not affect root Al level, thus alleviating *Citrus* Al-toxicity [6-7, 23]. Thus, the Al-induced increase in root Al accumulation and decrease in leaf and stem Al accumulation (Figure 8) might be an adaptive strategy of *C. sinensis* to Al-toxicity. However, the increase in Al-tolerance due to the increased pH could not be explained in this way, because Al distribution in roots of the Al-treated seedlings was higher at pH 2.5-3.0 than that at pH 3.5-4.0. We found that increasing the nutrient solution pH from 2.5 to 4.0 decreased the level of Al in roots, stems and leaves and the uptake of Al per root DW (Figure 5, 7), which might play a key role in the increased pH-induced alleviation of *Citrus* Al-toxicity.

### *4.3. Increased Uptake and Levels of Nutrients Might Play a Role in the Elevated pH-induced Alleviation of Al-toxicity*. Micromolar concentration of Al^3+^ can lead to a rapid inhibition of root growth, and subsequently interfering with the uptake of nutrients [2, 20]. Al decreased the uptake of N, P, K, Ca, Mg and Cu per plant at each given pH, especially at low pH with the exception that the uptake of Cu per plant was not altered by Al at pH 4.0. The uptake of S, B, Mn and Zn per plant were increased and decreased by Al at pH 3.5-4 and pH 2.5-3, respectively (Figure 7). Regression analysis indicated that both whole plant DW and leaf CO_2_ assimilation decreased with decreasing uptake of N, P, K, Ca, Mg, S, B or Fe per plant (Figures S5-S6). Generally viewed, Al decreased the levels of N, P, K, Ca, Mg and S in roots, stems and leaves, especially at pH 2.5-3.0 with the exceptions of a few, but increased the level of B in leaves, stems and roots at each given pH. Also, B level in roots, stems and leaves increased with increasing pH with or without Al-toxicity (Figures 5-6). Regression analysis showed that leaf CO_2_ assimilation decreased with decreasing leaf concentration of N, K, Ca, Mg, Fe or Zn (Figure S6). The supply of Ca, Mg, S, B and P can ameliorate Al-toxicity of plants [6-7, 23, 30-35]. PCA showed that N, P, K, Ca, Mg, S and B uptake per plant and/or per root DW were the main contributors to PC1 in the 0 mM Al-, 1 mM Al-, pH 4.0-, pH 3.5-, pH 3,0- and/or pH 2.5-treated seedlings (Tables S1-S6), demonstrating that the uptake of these elements might play a role in *Citrus* Al-toxicity (tolerance) and/or low pH-toxicity (tolerance). Thus, both the increased uptake per plant and/or root, stem and leaf levels of N, P, Ca, K, Mg, S and B might be involved in the increased pH-induced alleviation of *Citrus* Al-toxicity.

### *4.4. Causes for the Elevated pH-induced Alleviation of Photosynthetic Decline in the Al-treated Leaves.* Although g_s_ in the Al-treated leaves increased with increasing pH (Figure 2b) and leaf CO_2_ assimilation increased with increasing g_s_ (Figure S2), the ameliorative action of the increased pH against the inhibitory effect of Al on photosynthesis (Figure 2a) could not explained by the increased g_s_ alone, because low pH increased or did not affect both C_i_ and C_i_/C_a_ ratio (Figure 2) and leaf CO_2_ assimilation decreased with increasing C_i_ or C_i_/C_a_ ratio (Figure S2).

Previous studies showed that the impaired whole photosynthetic electron transport chain from the donor side of PSII to the reduction of PSI end acceptors was the main cause contributing to the Al-induced inhibition of photosynthesis in *Citrus* leaves [27], and that the Al-induced impairment of the whole photosynthetic electron transport chain and the subsequent decline in leaf CO_2_ assimilation could be alleviated by the supply of B, S and P [6-7, 23]. We observed that Al-toxicity lowered F_v_/F_m_ (a good indicator of photoinhibitory effects on PSII) and ψ_Eo_ (ET_o_/TR_o_), increased DI_o_/RC, and altered greatly OJIP transients in low pH treated leaves (Figures 3-4), together demonstrating that photoinhibition occurred in these leaves [36-37]. Increasing the nutrient solution pH from 2.5 to 4.0 prevented the Al-induced alterations of OJIP transients and all the 21 fluorescence parameters (Figures 3-4 and S3). Regression analysis showed that there was a positive relationships between leaf CO_2_ assimilation and F_m_, F_v_, F_v_/F_o_, F_OEC_, maximum amplitude of IP phase, F_v_/F_m_, ET_o_/ABS, RE_o_/ABS, ET_o_/TR_o_, RE_o_/TR_o_, ET_o_/RC, RE_o_/RC, RE_o_/CS_o_ or PI_abs,total_, but a negative relationship between leaf CO_2_ assimilation and F_o_, M_o_, DI_o_/ABS, ABS/RC, DI_o_/RC, TR_o_/RC or DI_o_/CS_o_ (Figure S4). These results suggested that the increased pH alleviated the Al-toxic impairment on the whole electron transport chain, thus preventing the Al-induced inhibition of photosynthesis.

### Evidence shows that the deficiencies of mineral nutrients (N, P, K, Ca and Mg) can impair the whole photosynthetic electron transport chain and cause a marked decline in leaf photosynthesis [38-43]. Here, the increased pH-induced alleviation of the Al-induced decreases in leaf levels of N, P, K, Ca and Mg and their uptake per plant might be one of the causes for preventing the Al-induced decline in leaf CO_2_ assimilation, as indicated by the positive correlations between leaf CO_2_ assimilation and leaf level of N, K, Ca or Mg and uptake per plant of N, P, K, Ca or Mg (Figure S6). Previous study showed that Al-toxicity increased or did not affect B concentration in *Citrus grandis* roots, stem and leaves, but supply B alleviated the Al-induced impairment occurring in the whole photosynthetic electron transport chain and inhibition of photosynthesis [23]. Our results showed that B concentration in roots, stems and leaves and its uptake increased with increasing pH (Figures 6-7), and that leaf CO_2_ assimilation increased with increasing B uptake per plant and displayed an increased trend with increasing leaf B concentration (Figure S6). These results indicated that the increased pH-induced increase in B uptake per plant might contribute to the alleviation of photosynthesis inhibition in Al-treated leaves. The antagonistic action of the increased pH against the inhibitory effect of Al-toxicity on leaf CO_2_ assimilation might also involve the increased pH-induced a decrease in leaf Al concentration (Figure 5a), as indicated by the negative and significant relationship between leaf CO_2_ assimilation and Al concentration (Figure S6) and the negative or positive relationships between leaf CO_2_ assimilation and Chl a fluorescence parameters (Figure S4). Based on these results, we concluded that increasing the solution pH from 2.5 to 4.0 mitigated the Al-induced impairment occurring on the whole photosynthetic electron transport chain, thus preventing the Al-induced decline in CO_2_ assimilation *via* decreasing the level of Al and increasing the uptake per plant of elements (N, P, K, Ca, Mg and B) and their levels in leaves.

## 5. Conclusions

Our data clearly demonstrated that a synergism existed between low pH and Al, and that increasing the nutrient solution pH from 2.5 to 4.0 alleviated the Al-toxicity of *C. sinensis* seedlings. Increasing pH decreased Al uptake per root DW and its level in roots, stems and leaves, and increased N, P, K, Ca, Mg, S and B uptake per plant and their levels in roots, stems and leaves. This might account for the increased pH-induced alleviation of *Citrus* Al-toxicity.

## Data Availability

The data used to support the findings of this study are available from the corresponding author upon request.

## Conflicts of Interest

The authors declare that there is no conflict of interest regarding the publication of this paper.

## Funding Statement

This study was financially supported by the National Key Research and Development Program of China (2018YFD1000305), the National Natural Science Foundation of China (31772257), the Special Fund for Scientific and Technological Innovation of Fujian Agriculture and Forestry University (CXZX2016148 and CXZX20127232), and the earmarked fund for China Agriculture Research System (No. CARS-27).The funders had no role in the design of the study and collection, analysis, and interpretation of data and in writing the manuscript.

## Supplementary Materials

Figure S1: Effects of pH and Al interactions on growth of *C. sinensis* seedlings. Figure S2: CO_2_ assimilation in relation to (a) stomatal conductance (g_s_), (b) intercellular CO_2_ concentration (C_i_) and (c) ratio of intercellular to ambient CO_2_ concentration (C_i_/C_a_) in *C. sinensis* leaves. Figure S3: Effects of pH and Al interactions on the high-light-induced OJIP transients of dark-adapted *C. sinensis* leaves plotted on a logarithmic time scale (0.01 to 1000 ms). Figure S4: Leaf CO_2_ assimilation in relation to 21 Chl a fluorescence parameters. Figure S5: Element uptake per plant in relation to (a-l) whole plant DW and (m-x) root DW in *C. sinensis* seedlings. Figure S6: Leaf CO_2_ assimilation in relation to (a-l) leaf element concentrations and (m-x) element uptake per plant in *C. sinensis* seedlings. Figure S7: Leaf Al concentration in relation to 21 Chl a fluorescence parameters. Table S1: PCA for physiological parameters of 0 mM Al-treated *C. sinensis* seedlings. Table S2: PCA for physiological parameters of 1mM Al-treated *C. sinensis* seedlings. Table S3: PCA for physiological parameters of pH 4-treated *C. sinensis* seedlings. Table S4: PCA for physiological parameters of pH 3.5-treated *C. sinensis* seedlings. Table S5: PCA for physiological parameters of pH 3-treated *C. sinensis* seedlings. Table S6: PCA for physiological parameters of pH 2.5-treated *C. sinensis* seedlings

## References

[1] T. B. Kinraide, “Identity of the rhizotoxic aluminium species”, *Plant and Soil*, vol. 134, pp. 167-178, 1991.

[2] K. V. Kochian, “Cellular mechanisms of aluminum toxicity and resistance in plants”, *Annual Review of Plant Physiology and Plant Molecular Biology*, vol. 46, pp. 237-260, 1995.

[3] H. R. von Uexküll, and E. Mutert, “Global extent, development and economic-impact of acid soils”, *Plant and Soil*, vol. 171, pp. 1-15, 1995.

[4] J. H. Guo, X. J. Liu, Y. Zhang et al., “Significant acidification in major Chinese croplands”, *Science*, vol. 327, pp. 1008-1010, 2010.

[5] C. J. Graham, “The influence of nitrogen source and aluminium on growth and elemental composition of ‘Nemaguard’ peach seedlings”, *Journal of Plant Nutrition*, vol. 24, pp. 423-439, 2001.

[6] H. X. Jiang, N. Tang, J. G. Zheng, Y. Li, and L.S. Chen, “Phosphorus alleviates aluminum-induced inhibition of growth and photosynthesis in *Citrus grandis* seedlings”, *Physiologia Plantarum*, vol. 137, pp. 298-311, 2009.

[7] P. Guo, Q. Li, Y. P. Qi et al., “Sulfur-mediated-alleviation of aluminum-toxicity in *Citrus grandis* seedlings”, *International Journal of Molecular Sciences*, vol. 18, Article ID2570, 2017.

[8] M. Moustakas, G. Ouzounidou, and R. Lannoye, “Aluminum effects on photosynthesis and elemental uptake in an aluminum-tolerant and non-tolerant wheat cultivar”, *Journal of Plant Nutrition*, vol.18, pp. 669-683, 1995.

[9] W. E. Pereira, D. L. de Siqueira, C. A.Martínez, and M. Puiatti, “Gas exchange and chlorophyll fluorescence in four *Citrus* rootstocks under aluminium stress”, *Journal of Plant Physiology*, vol. 157, pp. 513-520, 2000.

[10] P.H. Peixoto, F.M. Da Matta, J. Cambraia, “Responses of the photosynthetic apparatus to aluminum stress in two sorghum cultivars”, *Journal of Plant Nutrition*, vol. 25, 821-832, 2002.

[11] X. Zhao, Q Chen, Y. Wang, Z. Shen, W. Shen, and X. Xu, “Hydrogen-rich water induces aluminum tolerance in maize seedlings by enhancing antioxidant capacities and nutrient homeostasis”, *Ecotoxicology and Environmental Safety*, vol. 144, pp. 369-379, 2017.

[12] Z. Li, F. Xing, and D. Xing, “Characterization of target site of aluminum phytotoxicity in photosynthetic electron transport by fluorescence techniques in tobacco leaves”, *Plant and Cell Physiology*, vol. 53, pp. 1295-1309, 2012.

[13] J. Moustaka, G. Ouzounidou, I. Sperdouli, and M. Moustakas, “Photosystem II is more sensitive than photosystem I to Al^3+^ induced phytotoxicity”, *Materials*, vol. 11, Article ID1772, 2018.

[14] T. B. Kinraide, P. R. Ryan, and L. V. Kochian, “Interactive effects of Al^3+^, H^+^, and other cations on root elongation considered in terms of cell-surface electrical potential”, *Plant Physiology*, vol. 99, pp. 1461-1468, 1992.

[15] Y. Yang, Z. H. Guo, Z. J. Ye et al., “Changes of rhizosphere pH and the relationship with Al-resistant of different Al-tolerant wheat (*Triticum aestivum* L.) under Al stress”, *Journal of Agro-Environment Science*, vol. 29, pp. 636-641, 2010.

[16] J. Degenhardt, P. B. Larsen, S. H. Howell, and L. V. Kochian, “Aluminum resistance in the *Arabidopsis mutant alr*-104 is caused by an aluminum-induced increase in rhizosphere pH”, *Plant Physiology*, vol. 117, pp. 19-27, 1998.

[17] P. Wang, S. Bi, L. Ma, and W. Han, “Aluminum tolerance of two wheat cultivars (Brevor and Atlas66) in relation to their rhizosphere pH and organic acids exuded from roots”, *Journal of Agricultural and Food Chemistry*, vol. 54, pp. 10033-10039, 2006.

[18] M. Yang, L. Tan, Y. Xu et al., “Effect of low pH and aluminum toxicity on the photosynthetic characteristics of different fast-growing *Eucalyptus* vegetatively propagated clones”, *PloS One*, vol. 10, Article IDe0130963, 2015.

[19] T. B. Kinraide, “Toxicity factors in acidic forest soils: attempts to evaluate separately the toxic effects of excessive Al^3+^ and H^+^ and insufficient Ca^2+^ and Mg^2+^ upon root elongation”, *European Journal of Soil Science*, vol. 54, pp. 323-333, 2003.

[20] J. Xia, N. Yamaji, T. Kasai, and J. F. Ma, “Plasma membrane-localized transporter for aluminum in rice”, *Proceedings of the National Academy of Sciences of the United States of America*, vol. 107, pp. 18381-18385, 2010.

[21] Z. Lin, and D. L. Myhre, “*Citrus* root growth as affected by soil aluminum level under field conditions”, *Soil Science Society of America Journal,* vol. 54, pp. 1340-1344, 1990.

[22] Y. Li, M. Q. Han, F. Lin et al., “Soil chemical properties, ‘Guanximiyou’ pummelo leaf mineral nutrient status and fruit quality in the southern region of Fujian province, China”, *Journal of Soil Science and Plant Nutrition*, vol. 15, pp. 615-628, 2015.

[23] H. X. Jiang, N. Tang, J. G. Zheng, and L. S. Chen, “Antagonistic actions of boron against inhibitory effects of aluminum toxicity on growth, CO_2_ assimilation, ribulose-1,5-bisphosphate carboxylase/oxygenase, and photosynthetic electron transport probed by the JIP-test, of *Citrus grandis* seedlings”, *BMC Plant Biology*, vol. 9, Article ID102, 2009.

[24] A. Long, J. Zhang, L. T. Yang et al., “Effects of low pH on photosynthesis, related physiological parameters and nutrient profile of *Citrus*”, *Frontiers in Plant Science*, vol. 8, Article ID 185, 2017.

[25] J. Zhang, Q. Li, Y. P. Qi et al., “Low pH-responsive proteins revealed by a 2-DE based MS approach and related physiological responses in *Citru*s leaves”, *BMC Plant Biology*, vol. 18, Article ID188, 2018.

[26] P. Guo, Y. P. Qi, Y. T. Cai et al., “Aluminum effects on photosynthesis, reactive oxygen species and methylglyoxal detoxification in two *Citrus* species differing in aluminum tolerance”, *Tree Physiolog*y, vol. 38, pp. 1548-1565, 2018.

[27] H. X. Jiang, L. S. Chen, J. G. Zheng, S. Han, N. Tang, and B. R. Smith, “Aluminum-induced effects on photosystem II photochemistry in *Citrus* leaves assessed by the chlorophyll a fluorescence transient”, *Tree Physiology*, vol. 28, pp. 1863-1871, 2008.

[28] J. M. Banks, “Continuous excitation chlorophyll fluorescence parameters: a review for practitioners”, *Tree Physiology*, vol. 37, pp. 1128-1136, 2017.

[29] P. B. Vose, and P. J. Randall, “Resistance to aluminum and manganese toxicities in plants related to variety and cation exchange capacity”, *Nature*, vol. 196, pp. 85-86, 1962.

[30] I. R. Silva, T. J. Smyth, D. W. Israel, C. D. Raper, and T. W. Rufty, “Magnesium is more efficient than calcium in alleviating aluminium rhizotoxicity in soybean and its ameliorative effect is not explained by the Gouy-Chapman-Stern model”, *Plant and Cell Physiology*, vol. 42, pp. 538-545, 2001.

[31] I. R. Silva, T. J. Smyth, D. W. Israel, C. D. Raper, and T. W. Rufty, “Magnesium ameliorates aluminum rhizotoxicity in soybean by increasing citric acid production and exudation by roots”, *Plant and Cell Physiology*, vol. 42, pp. 546-554, 2001.

[32] M. Reyes-Díaz, C. Meriño-Gergichevich, E. Alarcón, M. Alberdi, and W. J. Horst, “Calcium sulfate ameliorates the effect of aluminum toxicity differentially in genotypes of highbush blueberry (*Vaccinium corymbosum* L.)”, *Journal of Soil Science and Plant Nutrition*, vol. 11, pp. 59-78, 2011.

[33] M. Riaz, L. Yan, X. Wu et al., “Boron alleviates the aluminum toxicity in *trifoliate orange* by regulating antioxidant defense system and reducing root cell injury”, *Journal of Environmental Management*, vol. 208, 149-158, 2018.

[34] L. Yan, M. Riaz, X. Wu, C. Du, Y. Liu, and C. Jiang, “Ameliorative effects of boron on aluminum induced variations of cell wall cellulose and pectin components in trifoliate orange (*Poncirus trifoliata* (L.) Raf.) rootstock”, *Environmental Pollution*, vol. 240, 764-774, 2018.

[35] C. Meriño-Gergichevich, G. Ondrasek, M. Zovko, D. Šamec, M. Alberdi, and M. Reyes-Díaz, “Comparative study of methodologies to determine the antioxidant capacity of Al-toxified blueberry amended with calcium sulphate”, *Journal of Soil Science and Plant Nutrition*, vol. 15, 965-978, 2015.

[36] L. Force, C. Critchley, and J. S. van Rensen, “New fluorescence parameters for monitoring photosynthesis in plants. 1. The effect of illumination on the fluorescence parameters of the JIP-test”, *Photosynthesis Research*, vol. 78, pp. 17-33, 2003.

[37] K. Maxwell, and G. N. Johnson, “Chlorophyll fluorescence-a practical guide”, *Journal of Experimental Botany*, vol. 51, pp. 659-668, 2000.

[38] C. W. Bednarz, D. M. Moosterhuis, and R. D. Evans, “Leaf photosynthesis and carbon isotope discrimination of cotton in response to potassium deficiency”, *Environmental and Experimental Botany*, vol. 39, pp. 131-139, 1998

[39] L. Cheng, L. H. Fuchigami, and P. J. Breen, “The relationship between photosystem II efficiency and quantum yield for CO_2_ assimilation is not affected by nitrogen content in apple leaves”, *Journal of Experimental Botany*, vol. 52, 1865-1872, 2001.

[40] N. Tang, Y. Li, and L. S. Chen, “Magnesium deficiency-induced impairment of photosynthesis in leaves of fruiting *Citrus reticulate* trees accompanied by up-regulation of antioxidant metabolism to avoid photo-oxidative damage”, *Journal of Plant Nutrition and Soil Science*, vol. 175, pp. 784-793, 2012.

[41] L. Cheng, and L. H. Fuchigami, “Rubisco activation state decreases with increasing nitrogen content in apple leaves”, *Journal of Experimental Botany*, vol. 51, 1687-1694, 2000.

[42] Z. H. Lin, L. S. Chen, R. B. Chen, F. Z. Zhang, H. X. Jiang, and N. Tang, “CO_2_ assimilation, ribulose-1,5-bisphosphate carboxylase/oxygenase, carbohydrates and photosynthetic electron transport probed by the JIP-test, of tea leaves in response to phosphorus supply”, *BMC Plant Biology*, vol. 9, Article ID 4, 2009.

[43] P. V. Rangnekar, “Effect of calcium deficiency on the carbon metabolism in photosynthesis and respiration of tomato leaf”, *Plant and Soil*, vol. 42, pp. 565-583, 1975.
